# The influence of the design of mental rotation trials on performance and possible differences between sexes: A theoretical review and experimental investigation

**DOI:** 10.1177/17470218231200127

**Published:** 2023-10-13

**Authors:** Leonardo Jost, Petra Jansen

**Affiliations:** Faculty of Human Sciences, University of Regensburg, Regensburg, Germany

**Keywords:** Mental rotation, sex differences, test design, multiple choice

## Abstract

Sex differences in mental rotation performance are one of the largest in cognitive psychology. Men outperform women by up to 1 *SD* in psychometric mental rotation tests, but it is often neglected that there are no or only small sex differences for chronometric tests. As both tests are supposed to measure the same ability, we suspect some features of the tests themselves to affect sex differences in performance. Following a theoretical review of the test features, we evaluate the effects of the number of possible answer alternatives, whether they are presented as pairwise mirrored, and their interaction on sex differences in mental rotation performance. In an online experiment, 838 German-speaking participants, 421 women, 417 men, *M*_age_ =  42.58 (*SD* = 12.54) years, solved four blocks of mental rotation trials with two or eight alternatives, which were either pairwise mirrored or not. The results show that that the overall performance was lower for more alternatives and for mixed alternatives but not for their interaction. We could not determine explanations for sex differences as we did not observe meaningful sex differences at all. Possible reasons include the differences between men and women in age and education. This study suggests that the differences between tests affect performance. Sex differences, however, need more investigation, including possible effects and interactions of the test design, education, and age.

Mental rotation describes the cognitive ability to rotate objects or images in the mind. As a spatial ability, the performance in mental rotation tests shows strong links to STEM (Science, Technology, Engineering, and Mathematics) performance (e.g., Hausmann, 2014; Moè, 2016; Moè et al., 2018). Gender/sex differences in mental rotation performance are often described as one of the largest in cognitive psychology (Voyer et al., 1995). They are thus often interpreted as a possible reason for the gender gap in STEM fields. A better understanding of these sex/gender differences could in turn help in closing the gender gap. However, it is often neglected that these sex/gender differences are not observed for all mental rotation tests.

Two types of mental rotation tests are widely used: (1) chronometric tests (for brevity referred to as SM tests) based on the study of R. N. Shepard and Metzler (1971), where two objects are presented which are either “same” (rotated) or “different” (mirrored) and (2) psychometric tests (VK tests) based on the study of Vandenberg and Kuse (1978), where compared with one target, two out of four alternatives are rotated and the other two are mirrored or structurally different. Both tests are suggested to measure the same mental rotation ability and the results of different mental rotation tests do correlate with each other (Voyer et al., 2006). However, large performance differences favouring men are only detected in VK tests, whereas there are only small or no differences in SM tests between men and women (Peters & Battista, 2008). Recently, Jost and Jansen (2020) evaluated another test design (JJ tests), where one target is compared with two alternatives, which are mirrored to each other and thus one is rotated to the target and one is mirrored. Interestingly, sex differences were also not observed for this type of test (Jost & Jansen, 2022).

Research has focused on examining gender and/or sex differences in VK tests relating to biological factors such as hormones, menstrual cycle, and sexual orientation (Hausmann et al., 2000, 2009; Peters et al., 2007; Peters, Laeng, et al., 1995), social factors such as gender stereotypes and stimulus familiarity (Hausmann et al., 2009; Moè et al., 2021; Ruthsatz et al., 2014, 2015, 2017), cultural aspects (Jansen et al., 2016; Peters et al., 2006), education and academic background (Moè et al., 2021; Peters et al., 2006, 2007; Peters, Laeng, et al., 1995), emphasising the relevance of role models (Neuburger et al., 2013), or strategy selection (Hegarty, 2018; Heil & Jansen-Osmann, 2008; Toth & Campbell, 2019; Voyer et al., 2020).^1^ However, there exists no conclusive theory for the occurrence of these sex differences. As sex differences also seem to increase with age during childhood and adolescence, research has investigated possible causes such as sex-stereotyped activities, parental language, or spatial toys, but again with no conclusive results (Newcombe, 2020). Another question regards the distinction between gender and sex. Studies investigating biological factors would indicate sex differences, whereas social factors would suggest differences by gender. However, most participants in studies are cis-gender persons and these might be confounded.^2^

Many of the aforementioned studies indeed identified interactions with sex differences. However, it must be emphasised that all those studies only investigated the VK test, which typically produces sex differences. If these factors influenced the mental rotation process, which all tests are supposed to measure, the same performance difference between sexes should occur in chronometric tests as most of those factors are also applicable. Thus, the question remains, why one test design provokes these factors to manifest in performance differences by sex while the other design does not. Peters and Battista (2008) and Bors and Vigneau (2011) already note that these differences might not be related to differences in actual mental rotation ability, but, for example, to the switching between pairwise comparisons within one trial for VK tests or dwelling on details of the stimuli. However, research investigating the test design has been scarcer but has analysed effects of the existence of a time limit (e.g., Peters, 2005; Voyer, 2011), the answering format (two out of four choice vs. same/different, Titze et al., 2010), or the distractor configuration (Voyer & Hou, 2006). While a relaxation of time limits and some aspects of the distractor configuration have been found to reduce performance differences between sexes, neither can fully explain the differences between tests.

To understand when and how sex differences in mental rotation tests emerge and whether they are linked to mental rotation ability, it seems necessary to consider the differences between the tests. However, a systematic comparison of test features is still lacking and to date no theory or experimental investigation exists that can explain the varying occurrences of sex differences in performance between tests. Despite much research focusing on sex differences in mental rotation ability, it is still unclear whether they are actually attributed to mental rotation ability or spatial abilities in general. One reason for the missing explanation for sex differences in VK tests could be that they are not actually linked to mental rotation ability but other features of the test. We thus believe this to be of great importance moving forward, to systematically investigate effects of the test design on mental rotation performance and sex differences.

In a first step, we attempt to systematise features of mental rotation tests. Concurrently, we will revisit existing theories for sex differences in mental rotation performance due to test features and how they hold up for a comparison between tests. Subsequently, we will formulate new theories regarding sex differences in mental rotation test performance. Furthermore, we will experimentally investigate the basis of these theories.

## Part 1: theories regarding sex differences in performance and a comparison between mental rotation tests

In this chapter, we aim to analyse the properties of mental rotation test designs and provide an overview on the current state of research on their influence on sex differences in performance. We review existing explanations for sex differences in mental rotation performance, which involve features of the tests. We will not review explanations that do not consider test features, as these cannot explain the observed pattern of differing sex differences between tests. A problem, again, is that no systematic test concept exists. Tests have often been varied in multiple ways, often unrelated to the specific research question (such as variations of time limits for investigations of stimulus material), which makes it difficult to isolate effects of specific features. This once again underlines the importance of a review of features of the tests themselves.

The differences (and similarities) between mental rotation tests can be grouped into two parts. First, there are differences between the individual trials of each test. Second, there are overarching differences of the test design and organisation mostly unrelated to individual trials.

### Differences between individual trials of different mental rotation tests

**Figure 1.**
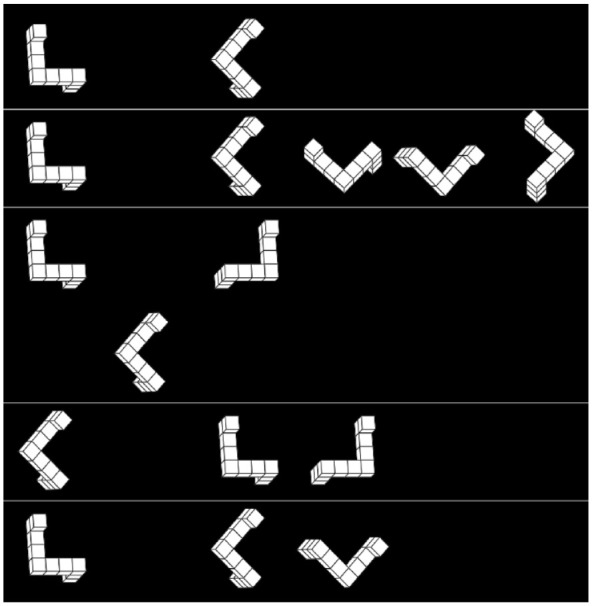
Different mental rotation trials. Exemplary trials from different mental rotation tests. From top to bottom: (1) SM trial; (2) VK trial; (3) JJ trial; (4) JJ trial in horizontal layout; and (5) JJ trial without paired choices/VK trial with only two choices. The respective tasks are to decide whether the two figures are the same or different (1) or to select exactly half of the alternatives, which are rotated versions of the separated target item to the left (2, 4, and 5) or bottom (3).

While the recent JJ test was inspired by the SM tests, the trials show a similarity to trials of VK tests, as always half of the answers are correct. In that way, it can be seen as a computerised type of VK trials with only two alternatives. Moreover, the two alternatives are aligned across their mirroring plane such that they are easily identifiable as mirrored. In VK trials the four alternatives are not easily pairwise identifiable as mirrored to each other. By modifying the layout of JJ trials to a horizontal positioning of all three figures, the differences and similarities between the tests are shown in Figure 1.

Thus, we propose three major differences between trials of the three mentioned mental rotation tests:

1. Compared with SM trials, participants are informed that always exactly half of the possible answers are correct in both JJ trials and VK trials. In most versions of the SM test, overall, about half of the trials are “same” trials and the other half “different” trials, but this is typically neither exact nor known to participants.
2. The trials use different number of alternatives (1, 2, or 4) and total number of items per trial (2, 3, or 5).
3. In JJ trials, the alternatives are pairwise identifiable as mirrored and aligned across the mirroring plane, whereas in VK trials, they are ordered randomly and not pairwise mirrored. For the SM trials, this distinction is not applicable.

In addition, there is a possible difference in the upright orientation of the figures. For abstract figures, such as the widely used cube figures and polygons, this is somewhat arbitrary as there is no clear upright orientation. While we can for the cube figures describe the upright orientation as an alignment with the natural axes (which are most of the time also the rotational axes), this is often not reported and might need further investigation. Another feature of the tests is the number of targets, but the used tests do not differ in this feature, as all use one target per trial.

Although the differences in the layout were necessary to highlight the differences between tests, they do not seem to play a major role in test performance. For SM trials, Xue et al. (2017) found no difference whether the left or the right item was rotated. For a version of VK trials with a vertically separated target, J. K. Krüger and Suchan (2016) found large sex differences in their control group, which was a sample of the general population. A comparison of the horizontal distances between stimuli mentioned by Battista and Peters (2010) also revealed no differences. Layout variations have been used without further inspection of the layout itself by Wohlschläger and Wohlschläger (1998), Wohlschläger (2001), and M. Krüger and Krist (2009) and different experiments regularly use varying computer screens and image sizes as well as non-standardised distances to the screen or test material and resulting optical distances between stimuli. Sex differences in VK tests and non-differences in SM tests have emerged under various of these non-systematically varied conditions.

Out of the suspected major differences, only the number of items per trial has yet been manipulated. Both Titze et al. (2008) and Voyer et al. (2020) restricted the visibility of the answer alternatives in VK tests. In the study of Titze et al., three out of four alternatives were hidden by a template and participants only saw the target and one alternative at a time. In two experiments, Voyer et al. employed a gaze-contingent display and participants could only see one item (either the target or one of the alternatives) at one time. However, these manipulations did not aim to change the number of alternatives and items within each trial, but only the visibility. To actually manipulate the number of items per trial, Titze et al. (2010) conducted a test that resembles a paper–pencil version of an SM test. Each trial of a VK test was split into four pairwise same/different judgements, which were then presented in random order. However, none of the manipulations could eliminate sex differences in performance. All three studies reported significantly better performance for men compared with women in all experiments, which are comparable with the performance differences of other VK tests. Titze et al. (2008) also found no reduction in sex differences compared with the regular test (the sex differences increased non-significantly from *d* = 0.40 to *d* = 0.45 by using the template).

### Differences between mental rotation tests

Next to the differences between individual trials of the tests, there are some overall differences between the tests. These were already partially manipulated by researchers, although not always as part of an analysis of sex differences. In the following, we describe some of these manipulations and their influence on sex differences. These studies do, however, often vary in more than the parameter in question (e.g., different time limits for digital tests compared with paper and pencil tests) and use different numbers of trials (we expect smaller standardised effect sizes of sex differences with less trials due to the increased relative variance of guessing).

#### Ending condition/time limits

In their most often used versions, the VK test is limited by both time and the number of trials (two times 3 min for 12 trials, Peters, Laeng, et al., 1995), the SM test is limited by the number of trials, and the JJ test is limited only by time. By relaxing or removing time limits in VK tests, sex differences in performance were reduced, but still easily detectable at Cohen’s *d* ≈ 0.5 and much larger than in SM tests (Peters, 2005; Voyer, 2011). As sex differences are neither commonly observed for SM tests limited by the number of stimuli and JJ tests limited by time, the ending conditions of tests cannot be the sole reason for sex differences.

#### Stimulus material

Whereas all tests most commonly use abstract cube figures as stimuli, there are small differences between the used figures. However, the original stimuli of Vandenberg and Kuse (1978) were based on the stimuli developed by R. N. Shepard and Metzler (1971) and redrawn versions of the stimuli have been used interchangeably between tests. For example J. K. Krüger and Suchan (2016) used the figures of Peters and Battista (2008), which are widely used in SM tests. Thus, other aspects of the stimuli and different stimuli have been investigated as possible reasons for sex differences in VK tests. The possible reasons include the embodiment of figures, gender stereotypes about the items, and the realism of figures. Moreover, the occlusions in the two-dimensional representations of three-dimensional objects have been analysed.

For human figures and a separation of partially occluded and nonoccluded figures, sex differences are still reported in VK tests, although often reduced for tests with overall higher average scores (e.g., Alexander & Evardone, 2008; Doyle & Voyer, 2013; Jansen & Lehmann, 2013; Voyer et al., 2020). As the widely used cube figures are seen as more male stereotyped, Ruthsatz et al. (2014, 2015, 2017) investigated female stereotyped stimuli and partially found reduced and even reversed sex differences in the VK test performance of children. These results have, however, only been partially transferred to adults with reduced or negated sex differences but no female advantage (Jost & Jansen, 2021; Rahe & Quaiser-Pohl, 2020; Rahe et al., 2020). Regarding the realism of stimuli, both Fisher et al. (2018) and Robert and Chevrier (2003) found no significant sex differences for real three-dimensional objects resembling cube figures. However, men were much faster than women in the study of Robert and Chevrier and scores approached the maximally attainable scores in the study of Fisher et al., indicating possible ceiling effects. While it is possible that more realistic figures reduce sex differences, no clear conclusions can be drawn as neither study was sufficiently powered to reliably detect smaller than large effects with only about 20 men and women per condition.

For SM tests, Voyer and Jansen (2016) did find better performance for men with human figures as stimuli.^3^ However, this is one of the few studies also reporting better performance for men for cube figures and the performance differences between stimuli were comparable for all stimulus types or even larger for the human figures (although quantified by differences in reaction time and accuracy on both same and different trials). Using hands as stimuli, Voyer, Jansen, et al. (2017) found better performance for men, whereas Campbell et al. (2018) detected possibly better performance for women. By comparing multiple stimulus types in SM tests, Jansen-Osmann and Heil (2007) found sex differences only for two-dimensional polygons. Heil and Jansen-Osmann (2008) replicated these sex differences for polygons and found larger differences for more complex polygons. Except for the polygons, all reported standardised effect sizes for sex differences in SM tests are small. As the cube figures show the largest reaction times and the lowest accuracies in comparisons of different stimuli and thus are the most complex in the study of Jansen-Osmann and Heil (2007), there does not seem to be a monotonous relationship between complexity and sex differences in SM tests. However, it is not clear whether the same effect occurs for VK tests as polygons have not yet been used as stimuli. Overall, it is likely that the used stimulus material and its complexity interacts with sex differences in mental rotation tests but that they are not a major reason for differences between different tests using cube figures.

#### The rotational axis

For many versions of VK tests only rotations in depth are used, whereas SM tests often use rotations in the picture plane or combine items rotated both in depth and in the picture plane in one test. In a study of a VK test with children, Ruthsatz et al. (2014) found an interaction of gender and axis: Larger sex differences occurred for cube figures rotated in depth compared with rotations in the picture plane. This effect was, however, not found for the second stimulus type, pellet figures. For adults, Battista and Peters (2010) did find better performance for rotations around a vertical axis in depth compared with a horizontal axis but no significant interaction with sex differences.

For SM tests using only rotations in depth, no or only small sex differences are found (Jordan et al., 2002; Peters, 2005; Rahe, Ruthsatz, Schürmann, & Quaiser-Pohl, 2019). The aforementioned sex differences for polygons are also only found for rotations in the picture plane. Many tests use multiple rotational axes as the widely used stimulus library of Peters and Battista (2008) provides stimuli rotated around all three canonical axes. The original study of R. N. Shepard and Metzler (1971) already suggested a strong similarity between rotational axes and these are often not further distinguished. For realistic stimuli, there is some evidence for better performance for rotations around a vertical axis compared with a horizontal rotation in picture plane (Foulkes & Hollifield, 1989). A possible reason could be the relevance of vertical rotations and gravity in real-life, but as Shiffrar and Shepard (1991) point out, these differences could be related to how the rotational axes are aligned with the features of the stimuli even for abstract figures. Despite the possibility for much further research into the effect of rotational axes, it seems unlikely that rotations in depth are a major reason for the discrepancy between sex differences found in different tests.

#### Type of distractors

Both SM tests and JJ tests use mirrored items as distractors, whereas VK tests also use structurally different items. By separating the answers for trials with mirrored and structural distractors in VK tests, mostly either non-significant effects of the distractor type or reduced sex differences and overall better performance for structural distractors are found (Boone & Hegarty, 2017; Bors & Vigneau, 2011; Doyle & Voyer, 2013; Monahan et al., 2008; Voyer & Hou, 2006). Thus, it seems that structural distractors in VK tests are likely not the reason for sex differences. However, Boone and Hegarty (2017) also analysed the incorporation of structural distractors with or without mirrored distractors in SM tests and found large sex differences, indicating that the combination with structural distractors is a possible reason for sex differences. But as they also found large sex differences on a VK test using only mirrored distractors in line with other uses of only mirrored distractors in VK tests (Rahe et al., 2020; Rahe & Quaiser-Pohl, 2020; Rahe, Ruthsatz, Jansen, & Quaiser-Pohl, 2019), their results only open this possibility to increase sex differences in SM tests but not how to reduce sex differences in VK tests.

#### Test administration

The VK tests were originally developed and are still often administered as a paper and pencil test, whereas SM and JJ tests are computerised to measure reaction times of individual trials. With the rising prevalence of computers since the original conception, many computerised and online versions of VK tests have been administered in more recent studies. These tests regularly report better performance for men, although sometimes with reduced effect sizes compared with paper and pencil tests (e.g., Debarnot et al., 2013; J. K. Krüger & Suchan, 2016; Monahan et al., 2008; Peters et al., 2007; Voyer et al., 2020). As mentioned before, in one study of a paper and pencil SM test, Titze et al. (2010) did find large sex differences in performance. While the test administration cannot be the sole reason for varying sex differences between mental rotation tests, the results of Titze et al. need to be investigated further.

#### Participant organisation

As with the test administration, the VK test was developed to quickly test large groups and multiple participants often perform the test in the same room at the same time. The group composition and stereotypes associated with genders have been hypothesised to influence performance differences (Moè, 2018). However, the comparison by Peters et al. (2006) of individual and group testing revealed no differences. In addition, but lacking systematic comparisons, associated with the form of the test administration, computerised and online versions of VK tests are often conducted individually or in the same manner as SM tests and still produce sex differences. Moreover, also individual testing using paper and pencil VK tests still produces sex differences (e.g., Titze et al., 2008).

#### Scoring system

To reduce the impact of guessing and based on the original work of Vandenberg and Kuse (1978), the most widely used scoring system for VK tests awards one point to a trial if and only if both correct alternatives are selected. These scores are not directly comparable to SM tests, for which the reaction time and accuracy is analysed for each “same” item and not analysed for “different” items. For JJ tests, there is no need to remove items, but reaction time and accuracy are still evaluated on a by-item basis. While participants are mostly instructed about the scoring system in VK tests, the task in SM tests is typically to solve the items as quickly and accurately as possible.

Due to the combination of multiple items into one trial, the reaction time in VK tests cannot be attributed to single items. For a comparison of scoring systems on VK tests, Voyer et al. (1995) found smaller, but still large sex differences for test scores on single items. For a comparison of both scoring systems within one test, Titze et al. (2010) found a high correlation and large sex differences for both. While VK tests bundle rotated items and distractors into one score, most SM tests only report scores for rotated items. In the few studies analysing both performance for mirrored stimuli and sex differences in SM tests, Peters (2005) observed no sex differences and Voyer and Jansen (2016) found small and comparable sex differences on both mirrored and non-mirrored trials. Kerkman et al. (2000) did find sex differences only for the mirrored trials, indicating a possible reason for the worse performance of women in VK tests. Their design, however, was different as very different stimuli were used and participants were also required to identify the direction of rotation. Moreover, their results indicate the tendency of women to guess that trials are non-mirrored (leading to higher accuracy for non-mirrored trials and lower accuracy on mirrored trials). While this led to sex differences only for the mirrored trials, this could also be interpreted as an overall worse performance. While the differences in the scoring systems prevent most results from being directly compared between tests, they may be a possible reason for reduced performance differences between sexes and could use some further investigation.

#### Practice trials and feedback

The mental rotation tests also differ in the number of practice trials before the results are recorded. While VK tests most commonly use 3 or 4 practice trials (composed of 12–16 alternatives), there is no standard for SM or JJ tests. The number of practice trials ranges from 0 to >100 with many studies using 10–20 practice trials comparable with VK tests, but there does not seem to be a systematic investigation of sex differences, depending on the amount of practice. Moreover, in repeated VK tests, sex differences are still found in later tests (Peters, 2005; Peters, Laeng, et al., 1995) despite the increased practice, although sometimes reduced (Peters, Chisholm, & Laeng, 1995). Another aspect is the inclusion of feedback for every trial during SM tests in some procedures. Rahe, Ruthsatz, Schürmann, et al. (2019) identified the missing feedback as a possible reason for sex differences. However, for example, both Jansen-Osmann and Heil (2007) and Voyer and Jansen (2016) used feedback for all trials and partially found sex differences.

### Theories on influences on sex differences in mental rotation tests

To conclude the comparison of mental rotation tests, there is no conclusive evidence or feature explaining the reasons for sex differences in mental rotation tests. Thus, new theories regarding the occurrence of the said sex differences are necessary.

#### A link between test difficulty and sex differences

One theory is that sex differences only emerge on tests with sufficient difficulty. A reoccurring pattern in the previous chapter is that sex differences in VK tests are typically reduced in test versions with higher scores (tests using simpler stimuli, easier identifiable distractors, increased available time, or digitalized tests). Still, sex differences are observed in VK tests but not or with smaller effect sizes in SM tests independent of these parameters. Moreover, even a combination of multiple factors reducing sex differences does not eliminate them. For example, Doyle and Voyer (2013, 2018) and the free-viewing experiment of Voyer et al. (2020) used computerised VK tests without time limits and human figures as stimuli and found better performance in men. Nevertheless, a more comprehensive comparison of mental rotation test scores seems necessary. Unfortunately, there is no direct way to compare scores between tests due to the different scoring systems and the lack of such a comparison once again points out the scarcity of comparisons between different mental rotation tests. We will thus attempt to provide some approximation of error rates for individual items in VK tests and a comparison with SM tests in the following.

Due to the information that always half of the alternatives are correct, the individual items are not independent. However, the exact dependency depends on the employed strategy and is impossible to estimate in general. In the following, we present two simple approximations of overall accuracy of single item comparisons in VK tests. These simple methods only estimate overall accuracies and do not account for differences between trials (e.g., lower accuracy with larger angular disparity). While a typical correction for multiple-choice tests concerns the number of answer alternatives (i.e., six possibilities to choose two out of four alternatives in VK tests), this is not necessary for a comparison between SM and VK tests. As we try to compute the probability that one single item in VK tests was solved correctly from the overall score, this includes the probability of guessing correctly in half of the cases. As a result, the corrected scores are directly comparable between tests as the probability to correctly identify rotated pairs, although they do not represent the true accuracy.

One way to estimate the probability p to solve a single item of a VK test correctly is to assume that trials are either solved by knowledge or guessed, that means the probability to solve a trial is either 1 or 1/6. Thus, 5/6 of the guessed trials are solved incorrectly.^4^ This means that the number of trials that were guessed correctly are 1/5 times the trials that were solved incorrectly. By transforming the guessing probability to chance level of half for single items, the resulting average probability is thus

$$\begin{array}{l} p_{1}=\left(\left(s-\frac{1}{5}\left(n-s\right)\right)+\frac{1}{2}\left(\frac{6}{5}\left(n-s\right)\right)\right)/n\\ \;\;\;\;\;\;\;\;\;\;=\left(s+\frac{2}{5}\left(n-s\right)\right)/n=\frac{3s}{5n}+\frac{2}{5} \end{array}$$

where s is the achieved score, n is the number of trials, and n−s is the number of trials solved incorrectly.

Another way to estimate single item accuracies is assuming the same probability for each item. This, however, is heavily dependent on the employed strategy. Assuming that a trial is solved correctly if three single item comparisons are solved correctly,^5^ the probability to solve a trial correctly is $s/n=p_{2}^{3}$ , that is, $p_{2}=\sqrt[{3}]{s/n}$ . For this simple measure, variance in $p_{2}$ is neglected. For a given $p_{2}$ , a variance between items within one trial would lead to lower scores than $p_{2}^{3}$ , whereas a variance between trials but not between items would lead to higher scores than $p_{2}^{3}$ . Note that $p_{2}>p_{1}$ for the range of interest ( $\frac{1}{6}<\frac{s}{n}<1$ ) as $p_{2}^{3}-p_{1}^{3}=0$ for $\frac{s}{n}=\sim -3.1,\sim 0.1,1$ .

We have compared both methods on all studies cited above which report the full values for accuracy of adult participants in VK tests in their standard form, which produce the most known and largest sex differences, and compared them with accuracies in SM tests. The full comparison is available in the supplementary material. Using either method, the overall accuracy for single task comparisons is estimated as .51 to .95. Assuming three to five single item comparisons are performed per trial (independent of the assumption for $p_{2}$ that of those only three are used to find the solution) and time limits of 3–6 min, these would be achieved at average reaction times of 1.5–5 s.^6^ Especially the male scores are thus comparable to accuracies in SM tests ranging from .6 to >.96 with most studies reporting accuracies of .85 or higher at average reaction times of 2–3 s. These typically only include rotated items, whereas reaction times are typically larger on mirrored trials. On the contrary, it is reported the accuracy on mirrored trials is mostly higher and also falls in the range of the estimated male VK performance. An exception to this is the study of Boone and Hegarty (2017), but they used multiple distractor types, which possibly increased the difficulty of distractor items but not of rotated items.

Based on these estimations, there do not seem to be sufficiently large differences in single item difficulty between tests to explain the large differences in sex differences. While reducing the overall difficulty of VK tests often reduces sex differences in performance, the non-differences in SM tests can likely not be explained by the difficulty of single items. Moreover, varying stimulus material in SM tests has rarely produced sex differences and these are not systematically related to task difficulty. Accuracy also drops and reaction time increases with larger angular disparity and sex differences have not consistently been observed with this increasing difficulty (see also Bors & Vigneau, 2011).

To conclude, it is unlikely that sex differences in VK tests are solely due to the increased difficulty of this test. It is, however, possible that increased difficulty amplifies sex differences or the detection of them due to the underlying binomial distribution of answers.

#### A link between trial design and sex differences

A second theory is that sex differences emerge due to features of the trial design, which differ between tests and have not been sufficiently investigated. These features are the number of alternatives per trial, the presentation of alternatives as pairwise mirrored, and, to an extent, the number of simultaneously presented trials and overall visible stimuli. These correspond to necessary and possible pairwise comparisons of stimuli to solve trials. In general, all mental rotation tests can be solved by repeated pairwise comparisons but differ in the number of necessary and possible pairwise comparisons to solve each trial. The overall number of possible pairwise comparisons between two figures are one for SM trials, three for JJ trials, and 10 for VK trials. Out of these, one, two, and four involve the target. Due to the information that exactly half of the alternatives are correct only one comparison for JJ trials and two or three comparisons for VK trials are necessary to complete the trial as the other items can then be solved by exclusion. Between computerised and paper–pencil tests, the tests also differ in the number of visible stimuli unrelated to the trial at hand as multiple trials are presented on one page. These offer further possible comparisons, which are not necessary and not helpful to find the solutions of individual trials. Either the number of necessary comparisons per trial, the number of possible comparisons per trial, the overall number of possible comparisons within a test, or a combination of all of them could be related to sex differences as all offer additional comparisons, which are not related to test performance.

However, neither of these effects nor their interaction has been conclusively investigated. Related to the comparisons within one trial, both the template task of Titze et al. (2008) and the restricted viewing experiments of Voyer et al. (2020) still required the same number of same/different judgements for one trial as the classical VK trials. However, in the trials of Titze et al., all pairwise comparisons involved the target. Regarding possible comparisons between trials, the paper–pencil SM test of Titze et al. (2010) had multiple and possibly related trials (same target) being visible at once.

The layout and visibility of stimuli could require spatial resources due to strategies of optimally disentangling the linked same/different judgements within one trial and the unlinked comparisons between different trials. The link to performance could thus be due to the spatial demands of the layout. The mediation study of Kaufman (2007) provides evidence that a large part of sex differences in VK tests is due to spatial working memory. In contrast, for a letter variation of the SM test, the dual-task study of Hyun and Luck (2007) indicates that what they called object working memory rather than spatial working memory is required to solve the task. However, due to the difference in designs and the rather small number of participants in the case of the study of Hyun and Luck, more research is needed to establish definitive links between working memory components and test versions. Next to possible spatial demands of the trial layout, both the number of comparisons necessary and the number of visible stimuli could be a measure of “perceived complexity.” Sex differences could then emerge due to different strategies between men and women for dealing with challenges due to, for example, speculated differences in upbringing and education (Määttä & Uusiautti, 2020).

#### Predictions by the theory

Thus, there are three possible predictions by the theory. The first possibility is that only the layout of the trials produces sex differences in tests. This may be due to the spatial demands of the layout and sex differences in spatial working memory. A consequence of this would be sex differences in favour of men in any test of other cognitive abilities using similar layout variations. The second and third possibilities suggest an interaction between the layout and the task. The second possible prediction is that the layout itself requires resources of a working memory subcomponent, likely the object or spatial working memory. If the task at hand requires resources of an overlapping working memory subcomponent and either of these working memory subcomponents has sex differences, then sex differences should be enhanced in the task performance. This would predict sex differences at least in tests of spatial abilities if a similar layout to VK tests is applied. To align this with the results of Hyun and Luck (2007), this would mean that either the layout requires resources of the object working memory, for which, however, a female advantage is observed (Voyer et al., 2007). Or the mental rotation task does require spatial working memory, just not to an extent that was exceeded in the dual task condition of Hyun and Luck and non-differences between sexes in SM tests are explained by superior object working memory of women similar to the observations of L. J. Levy et al. (2005). The third possibility is that the increased perceived complexity of the task due to the layout leads to sex differences if the added complexity of the tasks exceeds what women are confident to deal with more than for men. This would predict sex differences due to the trial layout for tests, which are already perceived as rather challenging.

## Part 2: experimental investigation of within-trial factors and sex differences in mental rotation performance

In any of the cases predicted by the theories discussed beforehand, sex differences should be enhanced in mental rotation tests due to the trial layout. Next to the possible underlying theories, the gathered evidence necessarily suggests that some difference between tests is a reason for varying sex differences. To better formulate, understand, and differentiate theories, it seems important to determine whether and which specific aspects of the trial layout produces or enhances sex differences. Due to the widely used different mental rotation tests, they thus are a prime candidate to identify these features.

Here, we aim to investigate the influence of the previously identified differences in the trial design on sex differences in test performance. The two most promising parameters seem to be the number of alternatives to each target and whether the alternatives are pairwise easily identifiable as mirrored to one another.

We will use a combination of paired or mixed alternatives with varying numbers of alternatives and the following hypotheses are investigated:

- We expect larger performance differences favoring men for the test using mixed alternatives.
- We expect larger performance differences favoring men for tests using more alternatives.
- Moreover, we expect the effect of the number of alternatives to be larger for the mixed alternatives.

As secondary hypotheses and due to the results of the pilot study, we predicted learning during the test and better performance in later blocks (Heil et al., 1998; Peters, 2005) and lower accuracies for larger angular disparities of items in line with common observations in SM tests. Moreover, we predicted lower overall performance for the mixed alternatives, for more alternatives, and for the interaction of both.^7^ The performance in later trials within a block will also be investigated, but the reduced performance in later trials observed in the pilot study contrasts the improvements observed during chronometric tests (Jost & Jansen, 2020).

### Pilot study

A preregistered first experiment was conducted using similar methods and investigating the same main hypotheses (the full method and results are also available at the preregistration and at Jost, 2022). However, due to an error in the a priori power analysis, the experiment was not sufficiently powered to detect all effect sizes of interest for the main hypotheses concerning effects between subjects. It should rather be treated as a pilot study. Nevertheless, there are some conclusions to improve the method for a second experiment and these will be presented briefly.

For the primary hypotheses, the number of alternatives were two, four, or eight. As secondary hypotheses, we also investigated the effects of additional covariates. Between subjects, STEM affiliation and previous experience with mental rotation were investigated. Within subjects, the angular disparity of trials and the learning within and between blocks were investigated.

The results provide some evidence that sex differences in mental rotation tests could be due to the trial layout, especially due to the mixed presentation of alternatives instead of a pairwise mirroring. The sex differences due to the trial layout interacted with both STEM education and previous experience. In line with the preregistered recruitment, the group of participants of main interest were those without previous experience with mental rotation. These, and especially the subgroup of non-STEM students, are the most tested sample in other studies, which provided the evidence for large sex differences. For these participants, the results show that sex differences are larger for mixed alternatives and smaller and not significant for paired alternatives in line with the hypothesis and the small or non-differences found for SM and JJ tests.

For the within-subjects effects, overall performance was lower for the mixed alternatives and for more alternatives. These effects also interacted for an even lower performance for more mixed alternatives. In line with the predictions, performance increased between blocks and decreased for larger angular disparities. Contrary to our prediction, performance decreased within blocks even when excluding unanswered trials due to the time limit.

Regarding the adaptation of the method for this experiment, the pilot study provides two insights. First, the overall effect of the number of alternatives was monotonic and roughly linear. This means employing more alternatives can enhance effects and this allows us to use larger differences in the number of alternatives to increase effect sizes and thus the power to detect differences. Second, the overall achieved scores are on average very similar to the assumed values in the power analysis and estimated in Table S1 (the average achieved scores ranging from .70 to .86 compared with the assumed values of .66–.81). The absolute differences between the scores of men and women were close to the expected values. However, the variance between participants was larger than expected and the expected large relative sex differences for VK tests (Cohen’s *d* > 0.7) for non-STEM participants without previous experience were only achieved for eight mixed alternatives and not for four mixed alternatives (Cohen’s *d* = 0.26). Possible reasons could be the larger variance in the sample due to recruitment from multiple universities or effects of the less controlled testing environment (one participant provided feedback that he would have preferred a mouse over a touchpad). Another reason for the lower relative sex differences could be the improvements between blocks and the usage of more blocks in this experiment compared with traditional VK tests, but also that only 24 items were used per condition instead of 48, which increases the relative variance of the binomial distribution. Whereas there are studies reporting also large sex differences for VK tests with only 12 trials (24 rotated items), these effects could be overestimated. While the estimated effects should not be overinterpreted (Brysbaert, 2019), the possibility of lower than previously estimated sex differences in performance and a lower correlation shall be considered in the following power analysis.

As we are also interested in explaining sex differences in VK tests found in the literature, we will focus on non-STEM affiliated people without extensive experience with mental rotation as these are the most studied population of interest. While the differential effects of STEM education and experience are still of interest, they exceed the scope of this work.

### Method

#### Power analysis

**Figure 2.**
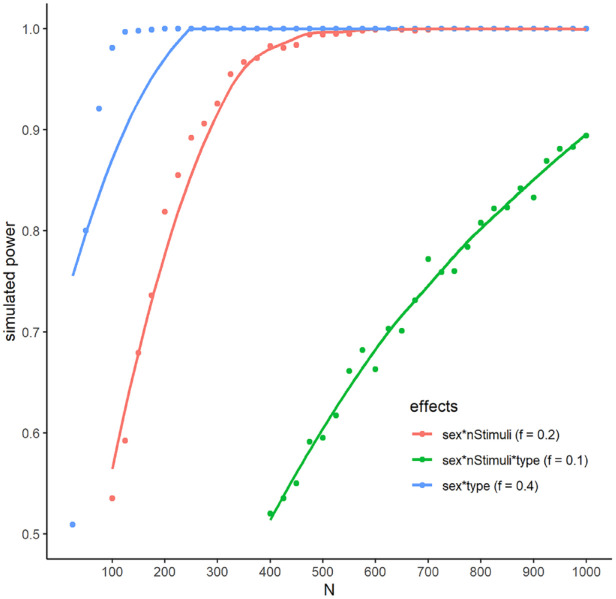
Simulated power for different numbers of participants using loess smoothing in ggplot2 (Wickham, 2016).

As a general guideline, Brysbaert (2019) suggests at least 860 participants to conclude that an effect is rather negligible at an effect size smaller than *d* = 0.2 for the comparison of two groups, which is a plausible lower bound here. As we are interested in finding all influencing factors on sex differences and explaining both sex differences and non-differences for the separate manipulations, this approach seems reasonable. An additional simulation was conducted to reach more specific estimates for the planned analyses. Based on the linearity of the effect of the number of alternatives observed in the pilot study and the theoretical considerations of differences between designs as well as the specifics of the planned tests using only 24 items per block, we estimated an overall difference of interest between two paired and eight mixed alternatives of the order of *d* = 0.8. Smaller overall sex differences would lead to harder to detect influencing factors, but would also point towards the influence of the trial design being smaller than expected. Moreover, for the conditions in between, we estimated the minimal values of interest of Cohen’s *f* for these interactions as 0.1, 0.2, and 0.4. The simulations suggested appropriate power of .8 at around 800 participants (see Figure 2). Thus, we opted for the suggestion of 860 participants at an equal distribution of men and women.

#### Participants

Participants were recruited through the online panels provided by Bilendi (www.meinungsplatz.de) and Respondi (mingle.respondi.de). The experiment targeted 430 adult men and 430 women without specific STEM affiliation and previous experience with mental rotation. Basic outlier detection was performed online such that these were not counted towards the final sample. The experiment was closed once 868 participations were logged for the final sample. Overall, 1,251 participations were logged at that time.

Participants were rewarded for their participation according to the guidelines of the panel providers. Before starting the study, all participants gave informed consent using a digital checkbox. Participants consented to anonymous sharing of their data and use of their data for scientific purposes. The experimental procedure was approved by the universities’ Ethics committee (Ethikkommission bei der Universität Regensburg, No. 20-2096_1-101).

**Figure 3.**
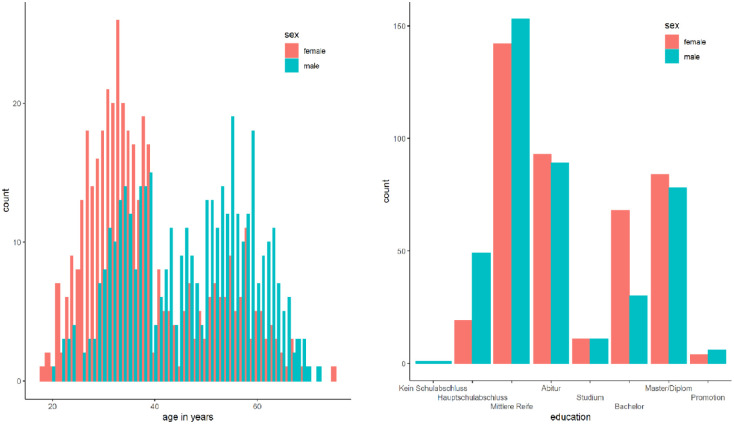
Distribution of age (left) and education (right) in the final sample.

Of the 838 participants included in the final sample, 421 were female and 417 were male. The mean age was 42.58 (*SD* = 12.54) years with men (46.85 ± 11.88) being older than women (38.36 ± 11.73); 5 women and 15 men had previous experience with mental rotation but neither acute nor chronic. Descriptively, the women were also better educated than men, with more women with university degrees (except for PhDs) and more men with lower school degrees. The distributions of age and education are presented in Figure 3 as they were used for further exploratory analyses.

#### Procedure

Participants were informed about the goals and procedure of the study before starting with a demographic questionnaire for prescreening purposes. Then, they completed the mental rotation tests succeeded by further questions about their experience with similar tests. Participants were asked to plan sufficient time (15–20 min) and complete the test alone. For purposes of monitoring participant numbers, there were two identical experiments. One was advertised to persons/households identified as female/women in the panel providers’ database and one to male/men.

#### Measures

##### Mental rotation

**Figure 4.**
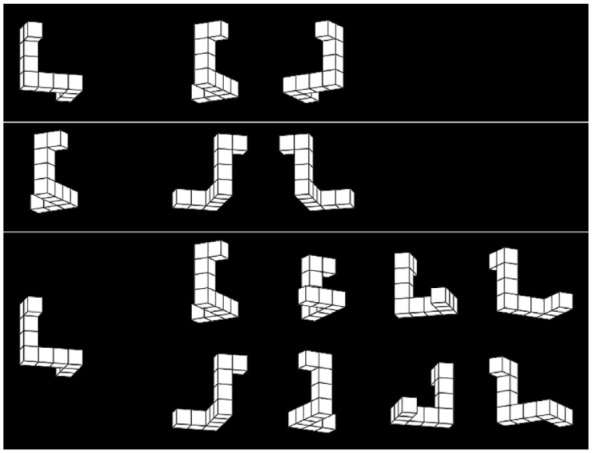
Examples of mental rotation trials with two mixed alternatives (top), two paired alternatives (centre), and eight mixed alternatives (bottom).

The mental rotation test was implemented using OSWeb (version 1.3.11) as part of OpenSesame (version 3.3.6; Mathôt et al., 2012) and made available online by JATOS (Lange et al., 2015). The test consisted of four blocks with a time limit of 3 min for each block. Each block consisted of trials of only one type of trials for a total of 48 alternatives (i.e., 24 trials with two alternatives or six trials with eight alternatives) in line with 12 trials for each block of VK tests. Trials were varied by the number of alternatives (two or eight) and by the pairing of alternatives (paired or mixed). Every participant completed one block for each combination. In the paired condition, the alternatives were pairwise horizontally mirrored to one another and aligned with the canonical axes while the target was rotated compared with the canonical axes. In the mixed condition, the target was aligned with the canonical axes and the alternatives were ordered randomly. For all numbers of alternatives, the target was on the left side of the screen and the alternatives were ordered from left to right with an additional space of 50px (at a resolution of 1,920 × 1,080) between the target and the first alternative. In the case of eight alternatives, there were two rows of four alternatives each. Examples of trials are shown in Figure 4.

The order of the blocks was randomised for each participant. Within each block for each participant, the used cube models and rotation angles for the target were randomised such that each combination of parameters occurred again only after all other choices had been presented at least once. The orientation (of the target and alternatives) and angles of the alternatives were chosen randomly such that no two figures were the same and all alternatives differed from the target by angle. All randomizations were performed using inline JavaScript in OpenSesame using the modern version of the Fisher–Yates algorithm.

The stimuli were generated using the library of Jost and Jansen (2020) using the parameters given in Table 1. Stimuli were rotated around the *z*-axis (vertical) first and the *x*-axis (horizontal) afterwards and the different orientations used different base angles such that the paired alternatives were actual mirror images of one another (see Figure 4). For the structure of the cube figures, Models 2–8 and 12–16 of the library of Peters and Battista (2008) were used as the other models can be transformed into these models by mirroring and/or rotating.

**Table 1.** Parameters for stimuli generation.

Parameter group	Parameter	Value
Colour options	Background colour	Transparent (black)
	Border colour	Black
	Face colour	White
Sizing and formatting	Cube diameter	42px
	Image size	340px × 340px
	File format	png
	Centering	Optical
Model properties	Base orientations	a, b
	Models	2–8 and 12–16 (Peters & Battista, 2008)
	Base rotation angles (*x*, *y*, *z*)	–15°,0°,15° (a), –15°,0°, –15°(b)
	Angle difference	45°
	Rotational axis	*z*
	Order of rotation	*z*, *x*

Participants could select and deselect the alternatives by clicking them with the mouse. Selected alternatives were marked by a quadratic white border. In the bottom-right corner of the screen, there was a button to continue with the next trial. The button could only be clicked once exactly half of the alternatives were selected. Otherwise, the button text asked the participants to select exactly half of the alternatives and could not be clicked. There was no option to return to previous trials.

The test was preceded by four practice trials in random order, one for each combination of two or eight alternatives and mixed or paired alternatives. For the practice trials, the participants had sufficient time (at most 15 min) and received feedback in the form of a red or green border around the selected alternatives.

Before each block, participants were instructed about the number of trials, the number of alternatives, and the time limit, but not about the pairing of alternatives. Between blocks, they were allowed a self-paced break. As in VK tests, participants were instructed that they would get one point if and only if they selected all correct alternatives. During the trials, participants could see the number of the current trial and the number of overall trials for the block in the top-left corner and the time left (since the start of the trial) in the top-right corner of the screen.

##### Demographics

A digital questionnaire was used to collect demographic information. Before the mental rotation test, participants were asked about their sex (male, female, or diverse), their age in years, their education (in eight levels ranging from “No high school diploma” to “PhD”), and their main field of work and education (from 18 possible categories). Participants were excluded at this stage if they selected one of the categories belonging to STEM fields (“Computer science,” “Engineering & Technology,” “Natural sciences & mathematics”).

After the mental rotation test, participants were asked about their previous experience with mental rotation (participants had to indicate if they had or had not participated in other mental rotation experiments with similar tasks before). Participants were excluded from analyses if they had experience with mental rotation tests in the past year (acute experience) or with more than three such tests (chronic experience).

#### Statistical analysis

Traditionally, mental rotation scores have been analysed using ANOVAs assuming a normal distribution despite the theoretically more correct binomial distribution for accuracy data. However, accuracy data for angular disparities in chronometric mental rotation tests as the only numerical predictor have regularly shown linear or less than linear decreases. If the underlying effect is assumed to be linear, these results contrast the predictions of the logit link function of the binomial distribution. For comparability with older work on sex differences in VK tests and due to the unclear linearity of effects, we thus used the normal distribution as the main analysis and provide a secondary analysis using the binomial distribution.^8^ For all analyses, we employed mixed effects models due to the advantages over traditional ANOVAs. For example, linear mixed models allow the simultaneous analysis of by-participant and by-item variances, thus eliminating the need to average over participants or items, while also facilitating the analysis of unbalanced data and achieving higher statistical power (Baayen et al., 2008; Barr et al., 2013; Hilbert et al., 2019).

The proportion of correctly solved items in each block was used as dependent variable and the number and pairing of alternatives, the sex, and their interaction were used as independent variables. For the secondary hypotheses, the position of the block was included as a predictor. For the binomial distribution, the angular disparity of items and the position of the trial within the block were used as additional predictors. In line with common scoring procedures, unattempted trials were treated as wrongly answered. For trials which were not finished due to the time limit, the answers were evaluated if at most half of the figures were selected. If more than half were selected, all answers to that trial were treated as unattempted.

Statistical analysis was performed with linear mixed models using MixedModels package (version 4.0.0; Bates et al., 2021) in Julia (version 1.6.2; Bezanson et al., 2017). Additional secondary analyses were performed using generalised mixed models with a binomial distribution. Model fit was calculated by using likelihood ratio tests to compare models with and without the effect of interest. Participants were used as random effects. Random slopes were selected stepwise starting with a maximal model and removing random slopes by dropping variance components using an LRT backwards heuristic at α = .2 (Matuschek et al., 2017). Non-significant fixed effects were further stepwise removed from the model, such that effects that least decreased model fits were removed first and a model containing only significant fixed effects remained. Non-significant effects were then tested for an improvement of model fit by inclusion in the resulting model, while significant effects were tested for worsening of model fit by exclusion of the effect. The resulting *p*-values were compared with a significance level of .05. The analysis of main effects contained in significant interactions was performed according to R. Levy (2014) and we thus used normalised sum contrasts for all categorical variables and centred all numerical variables and normalised them to a range of 1.

While there are several advantages to mixed models, the internal optimization procedures are not exact, which produces large imprecisions in both estimated effect sizes and *p*-values when the random effects are overparametrized. In addition to the procedures of Matuschek et al. (2017), we planned to reduce the random effects structures until these uncertainties were of magnitudes of .001 for the *p*-values and point estimates in our checked samples.

Four procedures were implemented to detect outliers. Participants were excluded if overall performance was below chance level (.5) on their attempted trials. This is the most often used outlier detection in mental rotation tests. However, performance of guessers should be symmetrically distributed around chance level and this procedure would thus only exclude about half of guessers. We thus implemented further outlier detection. These procedures were also designed to exclude participants who did not attempt to achieve the maximum score as instructed, as participants were given credit by the platform for simply completing the test, which may have incentivized participants to complete the tests quickly rather than actually trying to solve the tasks. Participants were thus excluded if the sum of their relative time used and their accuracy on their attempted trials was <1, which indicates a too strong focus on finishing the test quickly instead of accurately. These two outlier detection procedures were implemented online (albeit the online calculation of relative time did not account for the time limit and was thus less restrictive than the final outlier detection), such that these participants were not counted towards the desired sample size. Participants were further inspected as possible outliers if their overall accuracy was <.66 and they selected the first answer (leftmost alternative) in >90% of all trials or <10% of all trials indicating answering in a pattern instead of correctly. Participants who attempted less than half of all trials were also inspected for possible exclusion if their overall accuracy was <.66.

### Results

#### Outliers

**Figure 5.**
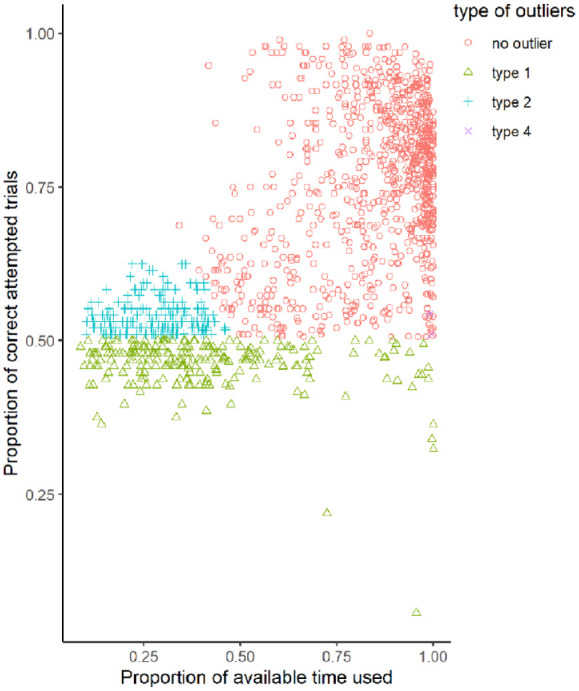
Speed–accuracy trade-offs and distribution of outliers.

Overall, 390 outliers had to be removed for performance reasons (216 men, 173 women, 1 diverse), 232 participants because of performance below chance level, an additional 156 participants for a too large focus on speed over accuracy, and two further participants with too few overall attempts (see Figure 5). Furthermore, 9 women and 14 men were excluded because of either acute or chronic experience with mental rotation tests.

#### Mental rotation data

**Figure 6.**
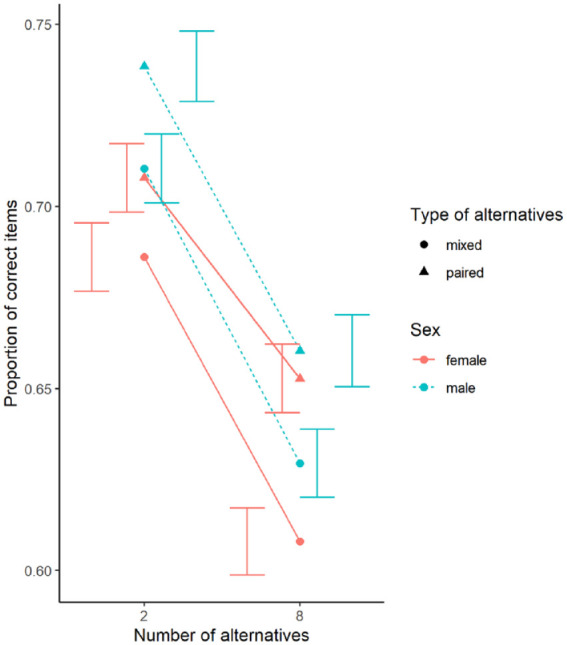
Proportion of correctly solved rotated items, separated by conditions and sex. Error bars represent standard error.

The overall proportion of correctly solved items per condition is shown in Figure 6. Overall Cohen’s *d* for the four conditions was 0.04 (8 paired), 0.11 (8 mixed), 0.13 (2 mixed), and 0.16 (2 paired alternatives).

The random effects structure of the mixed model included random slopes for the interaction and main effects of the number and type of alternatives as well as main effects for blocks and random intercepts. The main effects of the number of alternatives, the type of alternatives, and blocks were significant but neither interaction of interest was significant. Performance was better for less alternatives, for paired alternatives, and in later blocks. Men performed non-significantly better than women. The secondary alternative analysis using a binomial distribution yielded the same significance of the predictors. In addition, the angular disparity and the number of the trial within the block showed significant main effects. Performance was lower for larger angular disparities and for trials later within a block (see Table 2).

**Table 2.** Statistical analysis of proportion of correct items.

Variable	Estimate	*SE*	Test statistic	*p*
Normal distribution				
Intercept	0.67	0.01		
Type alternatives	–0.03	0.00	χ²(1) = 56.17	<.001
Number alternatives	–0.07	0.00	χ²(1) = 223.84	<.001
Sex	–0.02	0.01	χ²(1) = 3.59	.058
Number × sex	0.01	0.01	χ²(1) = 1.06	.303
Number × type	–0.01	0.01	χ²(1) = 2.41	.121
Sex × type	0.00	0.01	χ²(1) = 0.23	.632
Number × sex × type	–0.02	0.02	χ²(1) = 1.41	.235
Block	0.07	0.01	χ²(1) = 100.47	<.001
Binomial distribution				
Intercept	0.93	0.07		
Type alternatives	–0.18	0.02	χ²(1) = 58.04	<.001
Number alternatives	–0.47	0.03	χ²(1) = 260.13	<.001
Sex	–0.12	0.06	χ²(1) = 3.57	.059
Number × sex	0.05	0.05	χ²(1) = 0.90	.343
Number × type	–0.05	0.04	χ²(1) = 0.97	.325
Sex × type	–0.02	0.04	χ²(1) = 0.24	.626
Number × sex × type	–0.08	0.09	χ²(1) = 0.87	.352
Angular disparity	–0.34	0.03	χ²(1) = 127.56	<.001
Block	0.41	0.04	χ²(1) = 113.71	<.001
Trial no.	–0.79	0.03	χ²(1) = 394.81	<.001

*SE*: Standard error.

#### Exploratory analyses

**Figure 7.**
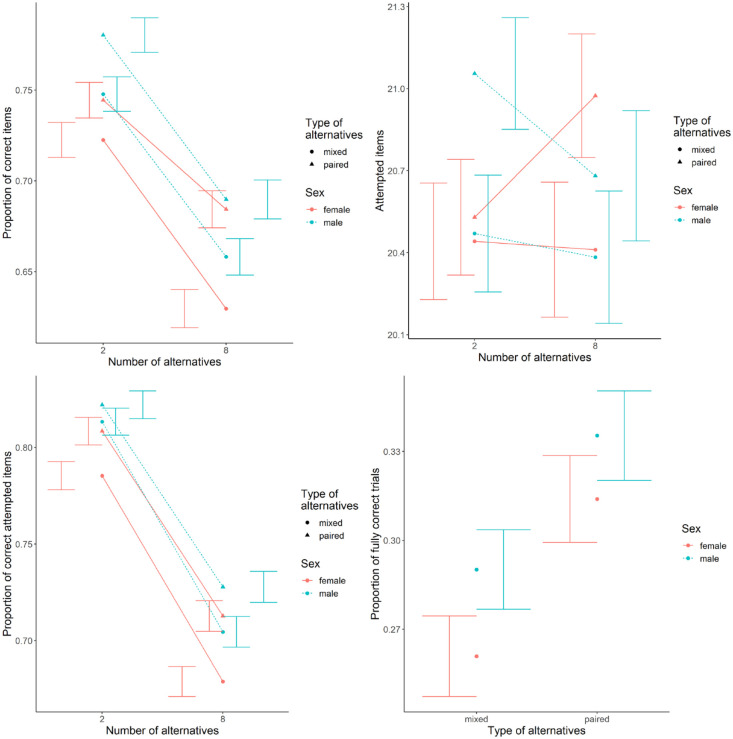
Descriptive data for the exploratory analyses using more restrictive outlier detection (top-left), the number of attempted items (top-right), the proportion of correctly solved attempted items (bottom-left), and the fully correctly solved trials (bottom-right). Error bars represent standard error.

Because of the unexpectedly small sex differences in all conditions compared with both the first experiment and the literature, the following variables were analysed in addition to the preplanned analyses. The descriptive data for these analyses is shown in Figure 7 and the statistical values in Table 3.

**Table 3.** Exploratory statistical analyses.

Variable	Estimate	*SE*	Test statistic	*p*
Age and education				
Age	–0.24	0.02	χ²(1) = 87.72	<.001
Education	0.04	0.02	χ²(1) = 3.12	.077
Sex	–0.06	0.01	χ²(1) = 26.11	<.001
Restrictive outliers				
Intercept	0.71	0.01		
Type alternatives	–0.03	0.00	χ²(1) = 55.87	<.001
Number alternatives	–0.09	0.01	χ²(1) = 238.27	<.001
Sex	–0.02	0.01	χ²(1) = 4.17	.041
Number × sex	0.01	0.01	χ²(1) = 0.89	.345
Number × type	–0.02	0.01	χ²(1) = 3.52	.061
Sex × type	–0.00	0.01	χ²(1) = 0.16	.692
Number × sex × type	–0.03	0.02	χ²(1) = 3.02	.082
Block	0.08	0.01	χ²(1) = 113.66	<.001
Attempted items				
Intercept	20.64	0.12		
Type alternatives	–0.40	0.09	χ²(1) = 18.88	<.001
Number alternatives	–0.09	0.12	χ²(1) = 0.53	.466
Sex	–0.09	0.24	χ²(1) = 0.13	.720
Number × sex	0.35	0.24	χ²(1) = 2.18	.140
Number × type	–0.09	0.18	χ²(1) = 0.26	.610
Sex × type	0.21	0.18	χ²(1) = 1.29	.255
Number × sex × type	–0.71	0.35	χ²(1) = 4.15	.042
Block	2.35	0.16	χ²(1) = 201.95	<.001
Accuracy on attempted items				
Intercept	0.76	0.00		
Type alternatives	–0.02	0.00	χ²(1) = 42.18	<.001
Number alternatives	–0.10	0.00	χ²(1) = 540.20	<.001
Sex	–0.02	0.00	χ²(1) = 5.90	.015
Number × sex	–0.00	0.01	χ²(1) = 0.01	.916
Number × type	–0.01	0.00	χ²(1) = 4.25	.039
Sex × type	–0.01	0.00	χ²(1) = 3.91	.048
Number × sex × type	0.00	0.01	χ²(1) = 0.08	.784
Block	0.01	0.01	χ²(1) = 1.00	.318
Proportion of fully correctly solved trials				
Intercept	0.30	0.01		
Type alternatives	–0.05	0.01	χ²(1) = 33.14	<.001
Sex	–0.03	0.02	χ²(1) = 2.28	.131
Sex × type	–0.01	0.02	χ²(1) = 0.30	.585
Block	0.04	0.01	χ²(1) = 11.09	<.001

*SE*: Standard error.

First, we looked at the effect of age and education because the women were younger and had higher degrees than the men and mental rotation performance was found to be higher for younger (for adults) and better educated persons (Peters et al., 2007). We included linear effects of age and education in ascending order. Both produced effects in the expected direction but the effect of education was only significant (*p* = .001) when not accounting for age. Controlling for these covariates also produced sex differences in overall performance, albeit the largest effect found when controlling for age was still smaller than expected (compared with the values in table S1) with men solving only about 6% more items correctly than women.

Second, because of the large number of outliers, we explored a more restrictive outlier detection by excluding all participants performing at or below 60% correct answers on their attempted trials (the mean between chance level and lowest expected accuracy scores of 70%). This procedure should include almost all participants who guessed but performed above chance level due to luck. An additional 136 participants (63 men, 71 women) were excluded. However, there was no visible or significant change in the patterns of the main hypotheses except for the small main effect of sex now being significant.

We furthermore looked at sex differences in the number of attempted items, the performance on only the attempted items, and for the trials using eight alternatives also the number of fully correctly solved trials in line with the traditional scoring system on VK tests. The number of attempted items showed some variation compared with the overall accuracy. The triple interaction of interest reached significance, but only after excluding non-significant effects. Moreover, participants attempted more of the paired trials compared with mixed trials, but the main effect of the number of alternatives was not significant. For the accuracy on only the attempted items, there was a significant sex difference, which also interacted with the type of alternatives. Despite these variations, sex differences for the accuracy on attempted items and for the fully correctly solved trials were of similar magnitude as for the overall correctly solved items.

## Discussion

Because sex differences are often assumed to be in mental rotation ability, the manipulation of VK tests, which produce the largest sex differences, is suspected as a means to uncover the reasons. However, the fact that sex differences do not occur or are much smaller on other tests of the same ability is often neglected. Thus, we have theoretically reviewed differences and similarities both overall and between individual trials of different mental rotation tests. Based on this comparison, it seems likely that the trial design is an important factor in the search for sex differences and we have experimentally investigated the number of alternatives and pairwise mirroring of alternatives.

The experiment provides evidence that the trial layout indeed influences the performance. The overall performance was lower for more alternatives and for mixed alternatives. There was, however, no evidence for the main hypotheses concerning varying sex differences between trial layouts. Given that we unexpectedly did not observe meaningful sex differences in overall performance, it is rather unsurprising that the search for explanations for such sex differences was fruitless. A major point of discussion must thus be whether sex differences in mental rotation tests exist at all or whether the non-detection of sex differences is related to our test or our sample. In addition, we also want to discuss the observed overall effects of the trial design and further results of the exploratory analyses as well as findings and future directions following from the theoretical review and comparison of tests.

### Sex differences in mental rotation and the relevance of the studied sample

To interpret the results as supporting or not supporting sex differences, we first need to establish the validity of the results. An issue of concern regarding our sample is the overall low performance and the large number of outliers. As Jost (2021) lined out, this could seriously reduce the interpretability of results. If participants were mostly guessing, the result could not be meaningfully related to performance and non-differences between men and women would not be surprising. In our case, almost one-third of the recorded participants were deemed outliers. With 230 of these performing below chance level as an indication of guessing, this would suggest that there are equally many guessers performing above chance level (due to the symmetry of the binomial distribution). The symmetry in the graphical inspection of the outliers suggests that many of the additional outliers were guessers performing above chance level, leaving around 100 guessers in the final dataset. This is still much more than observed in many other mental rotation studies, which may be due to the online nature, the recruitment, the incentive for participation, or the overall still rather low performance leading to more participants performing below chance level despite not guessing due to a binomial distribution of answers. However, even with this large number of outliers, the majority of participants should have performed the tasks correctly and the results should be interpretable. This is also supported by the additional more restrictive outlier detection, which should have excluded almost all guessers (at the cost of excluding more non-guessers) and produced the same pattern of results. Both the originally and more restrictively detected outliers also were roughly equally men and women, suggesting that regularly detected male performance advantages are not due to more (undetected) female guessers. Nevertheless, it should be noted that more guessers included in the sample should reduce overall effect sizes (because the effect size for guessers is 0).

Despite the low performance, it is thus now valid to discuss that we could not find the expected sex differences in performance. In most studies of mental rotation, participants are psychology students (or very similar), whereas our study targeted people from the general population without STEM affiliation. The final sample was less educated, with almost half of the participants not having the prerequisite school degrees for academic studies, and older than most university students. However, sex differences in mental rotation performance have not only been identified for students. Even online tests with large samples of the general population have identified a large male advantage contrary to the results presented here (J. K. Krüger & Suchan, 2016; Peters et al., 2007). One difference in the samples could be the exclusion of STEM affiliated persons in our study. As STEM affiliation is linked to better mental rotation performance (Hausmann, 2014; Moè et al., 2018; Peters, Laeng, et al., 1995), the larger proportion of men in STEM fields could be one cause of larger sex differences. This explanation, however, cannot explain the sex differences for university students and is thus unlikely to fully explain non-differences in performance. A further reason for the lower performance of women observed by J. K. Krüger and Suchan (2016) might be the fact that their control group excluded women with university degrees. Peters et al. (2007) did find that education was an important factor for mental rotation test performance even when accounting for age, which we could only partially replicate here. It must be noted that we only used a linear effect of education which surely cannot cover the differences between the recorded education levels and a possible nonlinear effect of overall education on performance. We also used different education levels compared with Peters et al. (2007) and their results suggest a nonlinear relationship. Regarding the age of participants, Peters et al. (2007) found a monotonous decrease in test performance for age groups >20 years, which was also supported by our data.

As we did observe sex differences in performance when accounting for age and education as well as when more restrictively excluding outliers,^9^ the sample characteristics in this study might be one key reason for us not finding overall performance differences and thus the difficulty in finding explanations for these. Our results can thus be interpreted as at least not contradictory to sex differences in mental rotation test performance in the general population. However, as the influence of the trial design on test performance could interact similarly for age, education, and sex, our results cannot provide evidence for or against the suggested explanations due to the trial design.

To conclude the discussion about our sample, it is necessary to search for explanations for sex differences on samples that actually produce sex differences. In the case of mental rotation, this means controlling especially for age, but possibly also for education, STEM affiliation, and previous experience with mental rotation. Moreover, the difficulty of the test should likely be adapted to the target sample to avoid flooring effects and the mixture of low performers and guessers as well as ceiling effects. This is already often done for children, but similar adaptations could be done for elder and less educated participants.

### The influence of the trial design

Despite our failure to gather meaningful evidence for the main hypotheses, we can draw some insights about the trial design from the results. While there is conflicting evidence for the interaction, both the pilot study and the final experiment provide evidence that performance decreases for mixed and for more alternatives. Thus, the complexity of the test is influenced not only by the task and the items but by the layout itself. In theory, all mental rotation tests should test the same mental rotation ability. The identified systematic variation of test performance, however, suggests that it is not that simple. Different trial layouts could tap differently into other areas such as working memory. One speculated difference could be a stronger involvement of spatial working memory in trials that require or allow more comparisons. Spatial working memory has shown involvement in VK tests (Kaufman, 2007) and a male advantage (Voyer, Voyer, & Saint-Aubin, 2017), which could thus explain male advantages in VK tests but not in SM or JJ tests.

The results also support common results of SM and JJ tests that accuracy decreases with angular disparity, which is interpreted as the involvement of mental rotation ability. The observed improvements between blocks also align with the same observation by Peters (2005), with improvements between blocks of SM tests (Heil et al., 1998), and between multiple VK tests (Meneghetti et al., 2017; Peters, Laeng, et al., 1995). For learning within blocks, however, our results are somewhat surprising. Despite controlling for unattempted trials at the end of blocks, we observed a decreasing performance. As we would have expected better performance due to learning between trials similar to the observed learning between blocks and within JJ tests (Jost & Jansen, 2020, 2022), this at least points to non-trivial effects of speed–accuracy trade-offs.

The exploratory analyses revealed some additional insights for the current dataset. Interestingly, significantly fewer items were attempted for the mixed alternatives compared with the paired alternatives but not for the trials with more alternatives compared with fewer alternatives. Despite both being harder as evidenced by the lower accuracy scores, participants took more time to reach their solutions only for the mixed alternatives. A possible reason could be the larger number of trials for fewer alternatives, including more breaks between trials and requiring more additional button clicks to proceed between trials. In another exploratory result, the effect of block was not significant for the accuracy of the attempted items. The increase in overall accuracy throughout the blocks was thus mainly due to more attempted items. These indicate, again, that effects of practice and the trial layout could influence speed–accuracy trade-offs and the perceived difficulty of items differently.

The detected small sex differences were also due to lower accuracy on attempted items and not fewer attempted items, which is contrary to the observation of significantly fewer attempted trials by women in the study of Peters (2005). On the contrary, these could explain the sex differences in psychometric tests without time limits. Given the small magnitude of sex differences observed here and the exploratory nature of these analyses, the transfer to the general population needs further confirmatory hypothesis testing.

### The influence of test difficulty

In the first part, we also discussed a possible effect of test difficulty. Although it seemed unlikely to be a sole explanation for sex differences, test difficulty may have had effects on both observed (non-) effects of sex differences and effects of trial design. First, sex differences are often enhanced at increased difficulty, and this may be both due to mathematical reasons (ceiling effects or the variance of the binomial distribution at different probabilities) or theoretical reasons (sex differences interact with test difficulty). In our case, it is unlikely that the test was too easy as average performance was even lower than expected and the number of outliers was higher than expected. Conversely, it is possible that the test was too difficult. In turn, it is possible that flooring effects rather than ceiling effects conceal the effects of interest. This is supported by the mathematical consideration that it should be increasingly difficult to lower performance the closer the performance gets to chance level, similar to increasing performance the closer the performance gets to full scores. This, however, is statistically not reflected in neither the binomial distribution nor the normal distribution used for analysis and the observation of effects might be further hindered by the larger relative variance of the binomial distribution the closer the probability gets to .5. These could have resulted in both the lower-than-expected sex differences as well as the conflicting results between this study and the pilot study regarding the effects of the interaction of the number and pairing of alternatives. As a side note, test difficulty is typically equated with inverse average performance, but these may not necessarily be the same.

### Theoretical review

Despite not being able to identify or refute reasons for sex differences in some mental rotation tests, the combination of the review of existing theories and the experimental results provides some insights as well as future directions. Most importantly, the experimental results suggest that performance is dependent on features of the trials. This means that widely used mental rotation tests measure more than just mental rotation ability. Because sex differences vary between tests, it is likely that the reason for sex differences lies in the aspects exceeding mental rotation ability. The proposed theories provide some possible explanations, but to further research it is necessary to first identify the exact test features and relatedly measured abilities and then, second, distinguish between possible theories. It must be noted that this may integrate all existing evidence explaining sex differences in VK tests independent of the consideration of test features. This evidence should, however, be related to the test and this may interact with mental rotation ability, but it should not be related to mental rotation ability in isolation.

Whereas much research has focused on reducing sex differences in VK tests, another interesting question is how to increase sex differences in tests. This could allow easier detection and thus more easily obtainable evidence to identify features or distinguish between theories. We have observed a monotonous effect of the number of alternatives on performance in general in the pilot study and this could be further employed in the search for sex differences, but there are further possibilities. Boone and Hegarty (2017) identified the incorporation of structural distractors to achieve sex differences in SM tests, but the removal of structural distractors in VK tests still produced large sex differences. Using polygons as stimuli has produced large sex differences even in SM test performance (Heil & Jansen-Osmann, 2008; Jansen-Osmann & Heil, 2007), but they have not yet been investigated for VK tests. It seems possible that polygons as stimuli would produce even larger sex differences in VK tests. Whereas polygons have been varied in complexity, similar approaches have not been employed for cube figures. They have only been compared with other item types despite the multiple possible modifications due to the abstract nature. While not directly contributing to reducing sex differences, exploring the effects of the trial design and the possibility to also increase sex differences could help us pinpoint the exact reasons and thus offer further understanding of the occurrence of sex differences in performance.

## Conclusion

In summary, we have reviewed and experimentally investigated the effect of the trial design on sex differences in mental rotation performance. The fact that sex differences vary strongly between different test versions has often been neglected in the search for explanations. Comparing the test versions has uncovered parameters of the trial designs, which were then evaluated experimentally. The results show that the overall performance depends on these parameters of the trial design. We, however, could not evaluate their effect on sex differences as we did not observe meaningful sex differences likely due to demographic differences in our sample.

## Outlook

Mental rotation is one special case of an ability where two tests are widely used, which both are supposed to measure the same ability, yet differ by quite a bit. Applying features, which have been found to reduce or produce sex differences in one test, to the other test could enhance our understanding of the occurrence of sex differences. Moreover, aspects identified to reduce sex differences in VK tests should be investigated regarding whether they simply reduce sex differences or instead increase female performance. Better understanding of the effects of the test design could also transfer to other cognitive tests.

Regarding the test design itself, we only investigated the features we deemed to be the most promising reasons for sex differences. Further potential trial and test manipulations include the upright orientation of figures, more complex rotations, the inclusion of multiple targets, or the presentation of multiple trials at the same time. On the contrary, we might have missed some features of VK tests in the tests employed in this study, which could potentially also be influential for sex differences.

## Supplemental Material

sj-docx-1-qjp-10.1177_17470218231200127 – Supplemental material for The influence of the design of mental rotation trials on performance and possible differences between sexes: A theoretical review and experimental investigation Supplemental material, sj-docx-1-qjp-10.1177_17470218231200127 for The influence of the design of mental rotation trials on performance and possible differences between sexes: A theoretical review and experimental investigation by Leonardo Jost and Petra Jansen in Quarterly Journal of Experimental Psychology

## Notes

1. But also see the criticism regarding interpretability of results for the study of Toth and Campbell (2019) by Jost (2021).

2. Although we are also not able to differentiate between gender and sex, we will refer to these as sex differences throughout this article for brevity.

3. Similar to different versions of cube figures, different versions of human figures have been used between different tests.

4. This does not account for trials in which one, two, or three items can be solved by knowledge, because the resulting probability depends heavily on the strategy.

5. One possible strategy is to solve items until two same or two different items are found and solving the remaining items by exclusion. If items were chosen randomly, there is a one-third probability that two items have to be solved and a two-third probability that three items have to be solved. However, if items are solved from left to right (or right to left), in the most used version of the test, only 2 out of 24 trials are solved correctly with only two items.

6. This comparison is independent of the number of finished tasks if one assumes that participants solve the same number of trials over time when trading speed for accuracy, but this does not hold for other possible speed–accuracy trade-offs (Liesefeld & Janczyk, 2019).

7. We note that we missed to state this prediction explicitly in the preregistration.

8. This analysis was preregistered as exploratory to explore the effects of distribution. To distinguish them from the exploratory analyses after inspection of the results, we call this secondary analysis here. For further reasoning, see also section O1 of the preregistration.

9. These factors might not be independent, as excluding outliers more restrictively could exclude more low-performing men due to higher age and lower education.

## References

[bibr1-17470218231200127] Alexander G. M. Evardone M. (2008). Blocks and bodies: Sex differences in a novel version of the Mental Rotations Test. Hormones and Behavior, 53(1), 177–184. 10.1016/j.yhbeh.2007.09.014 18036595 PMC2683583

[bibr2-17470218231200127] Baayen R. H. Davidson D. J. Bates D. M. (2008). Mixed-effects modeling with crossed random effects for subjects and items. Journal of Memory and Language, 59(4), 390–412. 10.1016/j.jml.2007.12.005

[bibr3-17470218231200127] Barr D. J. Levy R. Scheepers C. Tily H. J. (2013). Random effects structure for confirmatory hypothesis testing: Keep it maximal. Journal of Memory and Language, 68(3), 255–278. 10.1016/j.jml.2012.11.001 PMC3881361 24403724

[bibr4-17470218231200127] Bates D. Alday P. Kleinschmidt D. José Bayoán Santiago Calderón P. Zhan L. Noack A. Arslan A. Bouchet-Valat M. Kelman T. Baldassari A. Ehinger B. Karrasch D. Saba E. Quinn J. Hatherly M. Piibeleht M. Mogensen P. K. Babayan S. Gagnon Y. L. (2021). JuliaStats/MixedModels.jl: v4.0.0. Zenodo. 10.5281/zenodo.596435

[bibr5-17470218231200127] Battista C. Peters M. (2010). Ecological aspects of mental rotation around the vertical and horizontal axis. Journal of Individual Differences, 31(2), 110–113. 10.1027/1614-0001/a000020

[bibr6-17470218231200127] Bezanson J. Edelman A. Karpinski S. Shah V. B. (2017). Julia: A fresh approach to numerical computing. SIAM Review, 59(1), 65–98. 10.1137/141000671

[bibr7-17470218231200127] Boone A. P. Hegarty M. (2017). Sex differences in mental rotation tasks: Not just in the mental rotation process! Journal of Experimental Psychology: Learning Memory and Cognition, 43(7), 1005–1019. 10.1037/xlm0000370 28125253

[bibr8-17470218231200127] Bors D. A. Vigneau F. (2011). Sex differences on the mental rotation test: An analysis of item types. Learning and Individual Differences, 21(1), 129–132. 10.1016/j.lindif.2010.09.014

[bibr9-17470218231200127] Brysbaert M. (2019). How many participants do we have to include in properly powered experiments? A tutorial of power analysis with reference tables. Journal of Cognition, 2(1), 1–38. 10.5334/joc.72 31517234 PMC6640316

[bibr10-17470218231200127] Campbell M. J. Toth A. J. Brady N. (2018). Illuminating sex differences in mental rotation using pupillometry. Biological Psychology, 138, 19–26. 10.1016/j.biopsycho.2018.08.003 30086332

[bibr11-17470218231200127] Debarnot U. Piolino P. Baron J. C. Guillot A. (2013). Mental rotation: Effects of gender, training and sleep consolidation. PLOS ONE, 8(3), Article e60296. 10.1371/journal.pone.0060296 PMC3609807 23544134

[bibr12-17470218231200127] Doyle R. A. Voyer D. (2013). Bodies and occlusion: Item types, cognitive processes, and gender differences in mental rotation. Quarterly Journal of Experimental Psychology, 66(4), 801–815. 10.1080/17470218.2012.719529 22989237

[bibr13-17470218231200127] Doyle R. A. Voyer D. (2018). Photographs of real human figures: Item types and persistent sex differences in mental rotation. Quarterly Journal of Experimental Psychology, 71(11), 2411–2420. 10.1177/1747021817742079 30362408

[bibr14-17470218231200127] Fisher M. L. Meredith T. Gray M. (2018). Sex differences in mental rotation ability are a consequence of procedure and artificiality of stimuli. Evolutionary Psychological Science, 4(2), 124–133. 10.1007/s40806-017-0120-x

[bibr15-17470218231200127] Foulkes D. Hollifield M. (1989). Responses to picture-plane and depth mental-rotation stimuli in children and adults. Bulletin of the Psychonomic Society, 27(4), 327–330. 10.3758/BF03334617

[bibr16-17470218231200127] Hausmann M. (2014). Arts versus science: Academic background implicitly activates gender stereotypes on cognitive abilities with threat raising men’s (but lowering women’s) performance. Intelligence, 46(1), 235–245. 10.1016/j.intell.2014.07.004

[bibr17-17470218231200127] Hausmann M. Schoofs D. Rosenthal H. E. S. Jordan K. (2009). Interactive effects of sex hormones and gender stereotypes on cognitive sex differences-A psychobiosocial approach. Psychoneuroendocrinology, 34(3), 389–401. 10.1016/j.psyneuen.2008.09.019 18992993

[bibr18-17470218231200127] Hausmann M. Slabbekoorn D. Van Goozen S. H. M. Cohen-Kettenis P. T. Güntürkün O. (2000). Sex hormones affect spatial abilities during the menstrual cycle. Behavioral Neuroscience, 114(6), 1245–1250. 10.1037/0735-7044.114.6.1245 11142657

[bibr19-17470218231200127] Hegarty M. (2018). Ability and sex differences in spatial thinking: What does the mental rotation test really measure? Psychonomic Bulletin and Review, 25(3), 1212–1219. 10.3758/s13423-017-1347-z 28808983

[bibr20-17470218231200127] Heil M. Jansen-Osmann P. (2008). Sex differences in mental rotation with polygons of different complexity: Do men utilize holistic processes whereas women prefer piecemeal ones? Quarterly Journal of Experimental Psychology, 61(5), 683–689. 10.1080/17470210701822967 18421643

[bibr21-17470218231200127] Heil M. Rösler F. Link M. Bajric J. (1998). What is improved if a mental rotation task is repeated: The efficiency of memory access, or the speed of a transformation routine? Psychological Research, 61(2), 99–106. 10.1007/s004260050016 9689906

[bibr22-17470218231200127] Hilbert S. Stadler M. Lindl A. Naumann F. Bühner M. (2019). Analyzing longitudinal intervention studies with linear mixed models. TPM: Testing, Psychometrics, Methodology in Applied Psychology, 26(1), 101–119. 10.4473/TPM26.1.6

[bibr23-17470218231200127] Hyun J. S. Luck S. J. (2007). Visual working memory as the substrate for mental rotation. Psychonomic Bulletin and Review, 14(1), 154–158. 10.3758/BF03194043 17546746

[bibr24-17470218231200127] Jansen P. Lehmann J. (2013). Mental rotation performance in soccer players and gymnasts in an object-based mental rotation task. Advances in Cognitive Psychology, 9(2), 92–98. 10.2478/vl0053-008-0135-8 23833695 PMC3700661

[bibr25-17470218231200127] Jansen P. Zayed K. Osmann R. (2016). Gender differences in mental rotation in Oman and Germany. Learning and Individual Differences, 51, 284–290. 10.1016/j.lindif.2016.08.033

[bibr26-17470218231200127] Jansen-Osmann P. Heil M. (2007). Suitable stimuli to obtain (no) gender differences in the speed of cognitive processes involved in mental rotation. Brain and Cognition, 64(3), 217–227. 10.1016/j.bandc.2007.03.002 17433514

[bibr27-17470218231200127] Jordan K. Wüstenberg T. Heinze H. J. Peters M. Jäncke L. (2002). Women and men exhibit different cortical activation patterns during mental rotation tasks. Neuropsychologia, 40(13), 2397–2408. 10.1016/S0028-3932(02)00076-3 12417468

[bibr28-17470218231200127] Jost L. (2021). Concerns about cognitive performance at chance level. Scientific Reports, 11(1), 15530. 10.1038/s41598-021-93953-8 34330939 PMC8324882

[bibr29-17470218231200127] Jost L. (2022). Mental rotation: The test design, sex differences, and the link to physical activity. 10.5283/epub.51432

[bibr30-17470218231200127] Jost L. Jansen P. (2020). A novel approach to analyzing all trials in chronometric mental rotation and description of a flexible extended library of stimuli. Spatial Cognition & Computation, 20(3), 234–256. 10.1080/13875868.2020.1754833

[bibr31-17470218231200127] Jost L. Jansen P. (2021). Are implicit affective evaluations related to mental rotation performance? Consciousness and Cognition, 94(2021), 103178. 10.1016/j.concog.2021.103178 34343786

[bibr32-17470218231200127] Jost L. Jansen P. (2022). Manual training of mental rotation performance: Visual representation of rotating figures is the main driver for improvements. Quarterly Journal of Experimental Psychology, 75(4), 695–711. 10.1177/17470218211039494 PMC8915228 34344250

[bibr33-17470218231200127] Kaufman S. B. (2007). Sex differences in mental rotation and spatial visualization ability: Can they be accounted for by differences in working memory capacity? Intelligence, 35(3), 211–223. 10.1016/j.intell.2006.07.009

[bibr34-17470218231200127] Kerkman D. D. Wise J. C. Harwood E. A. (2000). Impossible “mental rotation” problems: A mismeasure of women’s spatial abilities? Learning and Individual Differences, 12(3), 253–269. 10.1016/S1041-6080(01)00039-5

[bibr35-17470218231200127] Krüger J. K. Suchan B. (2016). You should be the specialist! Weak mental rotation performance in aviation security screeners–reduced performance level in aviation security with no gender effect. Frontiers in Psychology, 7, Article 333. 10.3389/fpsyg.2016.00333 PMC4792886 27014142

[bibr36-17470218231200127] Krüger M. Krist H. (2009). Imagery and motor processes: When are they connected? The mental rotation of body parts in development. Journal of Cognition and Development, 10(4), 239–261. 10.1080/15248370903389341

[bibr37-17470218231200127] Lange K. Kühn S. Filevich E. (2015). “Just another tool for online studies” (JATOS): An easy solution for setup and management of web servers supporting online studies. PLOS ONE, 10(6), Article e0130834. 10.1371/journal.pone.0130834 PMC4482716 26114751

[bibr38-17470218231200127] Levy L. J. Astur R. S. Frick K. M. (2005). Men and women differ in object memory but not performance of a virtual radial maze. Behavioral Neuroscience, 119(4), 853–862. 10.1037/0735-7044.119.4.853 16187814

[bibr39-17470218231200127] Levy R. (2014). Using R formulae to test for main effects in the presence of higher-order interactions. arXiv:1405.2094. http://arxiv.org/abs/1405.2094

[bibr40-17470218231200127] Liesefeld H. R. Janczyk M. (2019). Combining speed and accuracy to control for speed-accuracy trade-offs(?). Behavior Research Methods, 51(1), 40–60. 10.3758/s13428-018-1076-x 30022459

[bibr41-17470218231200127] Määttä K. Uusiautti S. (2020). Nine contradictory observations about girls’ and boys’ upbringing and education—The strength-based approach as the way to eliminate the gender gap. Frontiers in Education, 5, 1–9. 10.3389/feduc.2020.00134

[bibr42-17470218231200127] Mathôt S. Schreij D. Theeuwes J. (2012). OpenSesame: An open-source, graphical experiment builder for the social sciences. Behavior Research Methods, 44(2), 314–324. 10.3758/s13428-011-0168-7 22083660 PMC3356517

[bibr43-17470218231200127] Matuschek H. Kliegl R. Vasishth S. Baayen H. Bates D. (2017). Balancing Type I error and power in linear mixed models. Journal of Memory and Language, 94, 305–315. 10.1016/j.jml.2017.01.001

[bibr44-17470218231200127] Meneghetti C. Cardillo R. Mammarella I. C. Caviola S. Borella E. (2017). The role of practice and strategy in mental rotation training: Transfer and maintenance effects. Psychological Research, 81(2), 415–431. 10.1007/s00426-016-0749-2 26861758

[bibr45-17470218231200127] Moè A. (2016). Does experience with spatial school subjects favour girls’ mental rotation performance? Learning and Individual Differences, 47, 11–16. 10.1016/j.lindif.2015.12.007

[bibr46-17470218231200127] Moè A. (2018). Effects of group gender composition on mental rotation test performance in women. Archives of Sexual Behavior, 47(8), 2299–2305. 10.1007/s10508-018-1245-0 29858725

[bibr47-17470218231200127] Moè A. Hausmann M. Hirnstein M. (2021). Gender stereotypes and incremental beliefs in STEM and non-STEM students in three countries: Relationships with performance in cognitive tasks. Psychological Research, 85(2), 554–567. 10.1007/s00426-019-01285-0 31960121

[bibr48-17470218231200127] Moè A. Jansen P. Pietsch S. (2018). Childhood preference for spatial toys. Gender differences and relationships with mental rotation in STEM and non-STEM students. Learning and Individual Differences, 68, 108–115. 10.1016/j.lindif.2018.10.003

[bibr49-17470218231200127] Monahan J. S. Harke M. A. Shelley J. R. (2008). Computerizing the Mental Rotations Test: Are gender differences maintained? Behavior Research Methods, 40(2), 422–427. 10.3758/BRM.40.2.422 18522051

[bibr50-17470218231200127] Neuburger S. Ruthsatz V. Jansen P. Heil M. Quaiser-pohl C. (2013). Acceptance and effects of role models in the spatial domain. Frontiers in Psychological and Behavioral Science, 2(3), 73–88.

[bibr51-17470218231200127] Newcombe N. S. (2020). The puzzle of spatial sex differences: Current status and prerequisites to solutions. Child Development Perspectives, 14(4), 251–257. 10.1111/cdep.12389

[bibr52-17470218231200127] Peters M. (2005). Sex differences and the factor of time in solving Vandenberg and Kuse mental rotation problems. Brain and Cognition, 57(2), 176–184. 10.1016/j.bandc.2004.08.052 15708213

[bibr53-17470218231200127] Peters M. Battista C. (2008). Applications of mental rotation figures of the Shepard and Metzler type and description of a mental rotation stimulus library. Brain and Cognition, 66(3), 260–264. 10.1016/j.bandc.2007.09.003 17967499

[bibr54-17470218231200127] Peters M. Chisholm P. Laeng B. (1995). Spatial ability, student gender, and academic performance. Journal of Engineering Education, 84(1), 69–73. 10.1002/j.2168-9830.1995.tb00148.x

[bibr55-17470218231200127] Peters M. Laeng B. Latham K. Jackson M. Zaiyouna R. Richardson C. (1995). A redrawn Vandenberg and Kuse Mental Rotations Test—Different versions and factors that affect performance. Brain and Cognition, 28(1), 39–58. 10.1006/brcg.1995.1032 7546667

[bibr56-17470218231200127] Peters M. Lehmann W. Takahira S. Takeuchi Y. Jordan K. (2006). Mental rotation test performance in four cross-cultural samples (N = 3367): Overall sex differences and the role of academic program in performance. Cortex, 42(7), 1005–1014. 10.1016/S0010-9452(08)70206-5 17172180

[bibr57-17470218231200127] Peters M. Manning J. T. Reimers S. (2007). The effects of sex, sexual orientation, and digit ratio (2D:4D) on mental rotation performance. Archives of Sexual Behavior, 36(2), 251–260. 10.1007/s10508-006-9166-8 17394056

[bibr58-17470218231200127] Rahe M. Quaiser-Pohl C. (2020). Cubes or pellets in mental-rotation tests: Effects on gender differences and on the performance in a subsequent math test. Behavioral Sciences, 10(1), 8–15. 10.3390/bs10010012 PMC7016669 31878056

[bibr59-17470218231200127] Rahe M. Ruthsatz V. Jansen P. Quaiser-Pohl C. (2019). Different practice effects for males and females by psychometric and chronometric mental-rotation tests. Journal of Cognitive Psychology, 31(1), 92–103. 10.1080/20445911.2018.1561702

[bibr60-17470218231200127] Rahe M. Ruthsatz V. Quaiser-Pohl C. (2020). Influence of the stimulus material on gender differences in a mental-rotation test. Psychological Research, 85, 2892–2899. 10.1007/s00426-020-01450-w 33237389

[bibr61-17470218231200127] Rahe M. Ruthsatz V. Schürmann L. Quaiser-Pohl C. (2019). The effects of feedback on the gender differences in the performance in a chronometric mental-rotation test. Journal of Cognitive Psychology, 31(4), 467–475. 10.1080/20445911.2019.1621872

[bibr62-17470218231200127] Robert M. Chevrier E. (2003). Does men’s advantage in mental rotation persist when real three-dimensional objects are either felt or seen? Memory and Cognition, 31(7), 1136–1145. 10.3758/BF03196134 14704028

[bibr63-17470218231200127] Ruthsatz V. Neuburger S. Jansen P. Quaiser-Pohl C. (2014). Pellet figures, the feminine answer to cube figures? Influence of stimulus features and rotational axis on the mental-rotation performance of fourth-grade boys and girls. In Lecture notes in computer science (Including subseries lecture notes in artificial intelligence and lecture notes in bioinformatics): Vol. 8684 LNAI (pp. 370–382). Springer. 10.1007/978-3-319-11215-2_26

[bibr64-17470218231200127] Ruthsatz V. Neuburger S. Jansen P. Quaiser-Pohl C. (2015). Cars or dolls? Influence of the stereotyped nature of the items on children’s mental-rotation performance. Learning and Individual Differences, 43, 75–82. 10.1016/j.lindif.2015.08.016

[bibr65-17470218231200127] Ruthsatz V. Neuburger S. Rahe M. Jansen P. Quaiser-Pohl C. (2017). The gender effect in 3D-Mental-rotation performance with familiar and gender-stereotyped objects–a study with elementary school children. Journal of Cognitive Psychology, 29(6), 717–730. 10.1080/20445911.2017.1312689

[bibr66-17470218231200127] Shepard R. N. Metzler J. (1971). Mental rotation of three-dimensional objects. Science, 171(3972), 701–703. 10.1126/science.171.3972.701 5540314

[bibr67-17470218231200127] Shiffrar M. M. Shepard R. N. (1991). Comparison of cube rotations around axes inclined relative to the environment or to the cube. Journal of Experimental Psychology: Human Perception and Performance, 17(1), 44–54. 10.1037//0096-1523.17.1.44 1826321

[bibr68-17470218231200127] Titze C. Heil M. Jansen P. (2008). Gender differences in the Mental Rotations Test (MRT) are not due to task complexity. Journal of Individual Differences, 29(3), 130–133. 10.1027/1614-0001.29.3.130

[bibr69-17470218231200127] Titze C. Heil M. Jansen P. (2010). Pairwise presentation of cube figures does not reduce gender differences in mental rotation performance. Journal of Individual Differences, 31(2), 101–105. 10.1027/1614-0001/a000018

[bibr70-17470218231200127] Toth A. J. Campbell M. J. (2019). Investigating sex differences, cognitive effort, strategy, and performance on a computerised version of the Mental Rotations Test via eye tracking. Scientific Reports, 9(1), 19430. 10.1038/s41598-019-56041-6 31857671 PMC6923419

[bibr71-17470218231200127] Vandenberg S. G. Kuse A. R. (1978). Mental rotations, a group test of three-dimensional spatial visualization. Perceptual and Motor Skills, 47(2), 599–604. 10.2466/pms.1978.47.2.599 724398

[bibr72-17470218231200127] Voyer D. (2011). Time limits and gender differences on paper-and-pencil tests of mental rotation: A meta-analysis. Psychonomic Bulletin and Review, 18(2), 267–277. 10.3758/s13423-010-0042-0 21327340

[bibr73-17470218231200127] Voyer D. Butler T. Cordero J. Brake B. Silbersweig D. Stern E. Imperato-McGinley J. (2006). The relation between computerized and paper-and-pencil mental rotation tasks: A validation study. Journal of Clinical and Experimental Neuropsychology, 28(6), 928–939. 10.1080/13803390591004310 16822733

[bibr74-17470218231200127] Voyer D. Hou J. (2006). Type of items and the magnitude of gender differences on the Mental Rotations Test. Canadian Journal of Experimental Psychology, 60(2), 91–100. 10.1037/cjep2006010 17133885

[bibr75-17470218231200127] Voyer D. Jansen P. (2016). Sex differences in chronometric mental rotation with human bodies. Psychological Research, 80(6), 974–984. 10.1007/s00426-015-0701-x 26358053

[bibr76-17470218231200127] Voyer D. Jansen P. Kaltner S. (2017). Mental rotation with egocentric and object-based transformations. Quarterly Journal of Experimental Psychology, 70(11), 2319–2330. 10.1080/17470218.2016.1233571 27603274

[bibr77-17470218231200127] Voyer D. Postma A. Brake B. Imperato-McGinley J. (2007). Gender differences in object location memory: A meta-analysis. Psychonomic Bulletin & Review, 14(1), 23–38. 10.3758/BF03194024 17546728

[bibr78-17470218231200127] Voyer D. Saint-Aubin J. Altman K. Doyle R. A. (2020). Sex differences in tests of mental rotation: Direct manipulation of strategies with eye-tracking. Journal of Experimental Psychology: Human Perception and Performance, 46(9), 871–889. 10.1037/xhp0000752 32324034

[bibr79-17470218231200127] Voyer D. Voyer S. D. Bryden M. P. (1995). Magnitude of sex differences in spatial abilities: A meta-analysis and consideration of critical variables. Psychological Bulletin, 117(2), 250–270. 10.1037/0033-2909.117.2.250 7724690

[bibr80-17470218231200127] Voyer D. Voyer S. D. Saint-Aubin J. (2017). Sex differences in visual-spatial working memory: A meta-analysis. Psychonomic Bulletin and Review, 24(2), 307–334. 10.3758/s13423-016-1085-7 27357955

[bibr81-17470218231200127] Wickham H. (2016). ggplot2: Elegant graphics for data analysis. Springer.

[bibr82-17470218231200127] Wohlschläger A. (2001). Mental object rotation and the planning of hand movements. Perception and Psychophysics, 63(4), 709–718. 10.3758/BF03194431 11436739

[bibr83-17470218231200127] Wohlschläger A. Wohlschläger A. (1998). Mental and manual rotation. Journal of Experimental Psychology: Human Perception and Performance, 24(2), 397–412. 10.1037/0096-1523.24.2.397 9606108

[bibr84-17470218231200127] Xue J. Li C. Quan C. Lu Y. Yue J. Zhang C. (2017). Uncovering the cognitive processes underlying mental rotation: An eye-movement study. Scientific Reports, 7(1), 1–12. 10.1038/s41598-017-10683-6 28855724 PMC5577169

