# Supplementary material for: The influence of the design of mental rotation trials on performance and possible differences between sexes: A theoretical review and experimental investigation
Source: Q J Exp Psychol (Hove). 2023 Oct 13;77(6):1250–71. doi: 10.1177/17470218231200127 (PMC11103899; doi:10.1177/17470218231200127)
Supplement: sj-docx-1-qjp-10.1177_17470218231200127 – Supplemental material for The influence of the design of mental rotation trials on performance and possible differences between sexes: A theoretical review and experimental investigation [file sj-docx-1-qjp-10.1177_17470218231200127.docx]

# Supplementary Material for:

**The Influence of the Design of Mental Rotation Trials on Performance and Possible Differences Between Sexes: A Theoretical Review and Experimental Investigation**

Leonardo Jost ORC ID: 0000-0003-3508-4315

Petra Jansen ORC ID: 0000-0002-0973-2158

University of Regensburg

Author Note

Leonardo Jost, Faculty of Human Sciences, University of Regensburg;

Petra Jansen, Faculty of Human Sciences, University of Regensburg.

Correspondence concerning this article should be addressed to Leonardo Jost, Faculty of Human Sciences, University of Regensburg, Universitätsstraße 31, 93053 Regensburg, Germany.

Email: [Leonardo.jost@ur.de](mailto:Leonardo.jost@ur.de)

**Prior Dissemination.** The theoretical discussion and pilot study are in part already published as part of a PhD Thesis (Jost, 2022). The data of the experiment was presented at the ESCoP 2022 conference in Lille.

## Comparison of Accuracy in Mental Rotation Tests

We have compared both proposed methods to estimate single item accuracy in VK tests on all studies cited in the first part of the manuscript, which report the values for accuracy. We only estimated performance of adult participants in VK tests in their standard form, which produce the most known and largest sex differences, and compared them with accuracies in SM tests. These are VK tests with time limits of 3 or 6 min per 12 items, no time limits for trials, and all tests using cube figures. We added further studies from our personal database satisfying these conditions. For the comparison of mirrored trials, we included all studies known to us that report data on mirrored trials. This includes the study of Paschke et al. (2012) for which only graphical results are available. The estimated accuracies are shown in table S1 and the scores in SM tests are shown in table S2.

**Table S1**

*Estimated Single Item Accuracy From VK Test Scores Using the Proposed Calculation Methods.*

| Study | Score(n) | $p_{1}$ | $p_{2}$ |
| --- | --- | --- | --- |
| Alexander and Evardone (2008) block figures | Men 6.74 (12)  Women 3.85 (12)  Men ratio 0.71  Women ratio 0.48 | .74  .59  .83  .69 | .83  .68  .89  .78 |
| Battista and Peters (2010) horizontal axis | Men 9.7 (24)  Women 6.8 (24) | .64  .57 | .74  .66 |
| Battista and Peters (2010) vertical axis | Men 13.1 (24)  Women 8.9 (24) | .73  .62 | .82  .72 |
| Doyle and Voyer (2013) | Men ratio .65-.81  Women ratio .47-66 | .79-.89  .68-.80 | .87-.93  .78-87 |
| Jansen et al. (2016) | Men 6.13-13.91 (24)  Women 4.56-11.90 (24) | .55-.75  .51-.70 | .63-.83  .57-.79 |
| Moè (2018) | Men 8.83-10.00 (24)  Women 5.00-5.92 (24) | .62-.65  .52-.55 | .72-.75  .59-.63 |
| Moè et al. (2021) | Men 12.64 (24)  Women 8.64 (24) | .72  .62 | .81  .71 |
| Monahan et al. (2008) computer test | Men 12.34 (24)  Women 9.57 (24) | .71  .64 | .80  .74 |
| Monahan et al. (2008) paper and pencil test | Men 12.83 (24)  Women 8.15 (24) | .72  .60 | .81  .70 |
| Peters, Laeng et al. (1995) | Men 12.1-14.8 (24)  Women 8.2-10.4 (24) | .70-.77  .60-.66 | .80-.85  .70-.76 |
| Peters (2005) Study 1, 3 minutes | Men 12.6 (24)  Women 8.8 (24)  Men ratio 0.739  Women ratio 0.602 | .72  .62  .84  .76 | .81  .72  .90  .84 |
| Peters (2005) Study 2, 6 minutes | Men 18.0 (24)  Women 14.4 (24)  Men ratio 0.776  Women ratio 0.642 | .85  .76  .87  .79 | .91  .84  .92  .86 |
| Peters et al. (2006) | Men 11.2-16.6 (24)  Women 8.3-12.8 (24) | .68-.82  .61-.72 | .78-.88  .70-.81 |
| Rahe et al. (2019) | Men 5.74 (12)  Women 3.90 (12) | .69  .60 | .78  .69 |
| Titze et al. (2008) without pattern | Men 20.76 (24)  Women 18.91 (24) | .92  .87 | .95  .92 |

*Note.* The ratios for the study of Peters (2005) were calculated from the reported percentage of problems attempted.

**Table S2**

*Average Reaction Times and Accuracies in SM Tests Using Cube Figures.*

| Study | Reaction time (s) | Accuracy |
| --- | --- | --- |
| Adams et al. (2014) picture plane  Adams et al. (2014) depth | 4.7  5.2 | .80  .61 |
| Jansen-Osmann and Heil (2007) | 1.84 | .954 |
| Jolicœur et al. (1985) experiment 1 | 4.5 | .87 |
| Paschke et al. (2012)  Paschke et al. (2012) Mirrored trials | ~2.5  ~2.7 | ~.85  ~.95 |
| R.N. Shepard and Metzler (1971)  R.N. Shepard and Metzler (1971) mirrored trials | ~3  3.8 | .968 |
| S. Shepard and Metzler (1988)  S. Shepard and Metzler (1988) mirrored trials | 2.06 | .906  .942 |
| Voyer and Jansen (2016)  Voyer and Jansen (2016) mirrored trials | 2.10  2.52 | .874  .890 |
| Wiedenbauer et al. (2007) experiment 1 | ~2.6 | .882 |
| Wiedenbauer et al. (2007) experiment 2 |  | .876 |

*Note.* For intervention studies, pretest data is shown. For studies that estimate rotation speed, the reaction time is calculated for an angle of 90°. Values marked with ~ are estimated from figures.

# References

Adams, D. M., Stull, A. T., & Hegarty, M. (2014). Effects of Mental and Manual Rotation Training on Mental and Manual Rotation Performance. *Spatial Cognition and Computation*, *14*(3), 169–198. https://doi.org/10.1080/13875868.2014.913050

Alexander, G. M., & Evardone, M. (2008). Blocks and bodies: Sex differences in a novel version of the Mental Rotations Test. *Hormones and Behavior*, *53*(1), 177–184. https://doi.org/10.1016/j.yhbeh.2007.09.014

Battista, C., & Peters, M. (2010). Ecological aspects of mental rotation around the vertical and horizontal axis. *Journal of Individual Differences*, *31*(2), 110–113. https://doi.org/10.1027/1614-0001/a000020

Doyle, R. A., & Voyer, D. (2013). Bodies and occlusion: Item types, cognitive processes, and gender differences in mental rotation. *Quarterly Journal of Experimental Psychology*, *66*(4), 801–815. https://doi.org/10.1080/17470218.2012.719529

Jansen-Osmann, P., & Heil, M. (2007). Suitable stimuli to obtain (no) gender differences in the speed of cognitive processes involved in mental rotation. *Brain and Cognition*, *64*(3), 217–227. https://doi.org/10.1016/j.bandc.2007.03.002

Jansen, P., Zayed, K., & Osmann, R. (2016). Gender differences in mental rotation in Oman and Germany. *Learning and Individual Differences*, *51*, 284–290. https://doi.org/10.1016/j.lindif.2016.08.033

Jolicœur, P., Regehr, S., Smith, L. B. J. P., & Smith, G. N. (1985). Mental rotation of representations of two-dimensional and three-dimensional objects. *Canadian Journal of Psychology/Revue Canadienne de Psychologie*, *39*(1), 100–129. https://doi.org/10.1037/h0080118

Moè, A. (2018). Effects of Group Gender Composition on Mental Rotation Test Performance in Women. *Archives of Sexual Behavior*, *47*(8), 2299–2305. https://doi.org/10.1007/s10508-018-1245-0

Moè, A., Hausmann, M., & Hirnstein, M. (2021). Gender stereotypes and incremental beliefs in STEM and non-STEM students in three countries: relationships with performance in cognitive tasks. *Psychological Research*, *85*(2), 554–567. https://doi.org/10.1007/s00426-019-01285-0

Monahan, J. S., Harke, M. A., & Shelley, J. R. (2008). Computerizing the mental rotations test: Are gender differences maintained? *Behavior Research Methods*, *40*(2), 422–427. https://doi.org/10.3758/BRM.40.2.422

Paschke, K., Jordan, K., Wüstenberg, T., Baudewig, J., & Leo Müller, J. (2012). Mirrored or identical - Is the role of visual perception underestimated in the mental rotation process of 3D-objects?: A combined fMRI-eye tracking-study. *Neuropsychologia*, *50*(8), 1844–1851. https://doi.org/10.1016/j.neuropsychologia.2012.04.010

Peters, M. (2005). Sex differences and the factor of time in solving Vandenberg and Kuse mental rotation problems. *Brain and Cognition*, *57*(2), 176–184. https://doi.org/10.1016/j.bandc.2004.08.052

Peters, M., Laeng, B., Latham, K., Jackson, M., Zaiyouna, R., & Richardson, C. (1995). A redrawn vandenberg and kuse mental rotations test - different versions and factors that affect performance. *Brain and Cognition*, *28*(1), 39–58. https://doi.org/10.1006/brcg.1995.1032

Peters, M., Lehmann, W., Takahira, S., Takeuchi, Y., & Jordan, K. (2006). Mental rotation test performance in four cross-cultural samples (N = 3367): Overall sex differences and the role of academic program in performance. *Cortex*, *42*(7), 1005–1014. https://doi.org/10.1016/S0010-9452(08)70206-5

Rahe, M., Ruthsatz, V., Jansen, P., & Quaiser-Pohl, C. (2019). Different practice effects for males and females by psychometric and chronometric mental-rotation tests. *Journal of Cognitive Psychology*, *31*(1), 92–103. https://doi.org/10.1080/20445911.2018.1561702

Shepard, R. N., & Metzler, J. (1971). Mental Rotation of Three-Dimensional Objects. *Science*, *171*(3972), 701–703. https://doi.org/10.1126/science.171.3972.701

Shepard, S., & Metzler, D. (1988). Mental Rotation: Effects of Dimensionality of Objects and Type of Task. *Journal of Experimental Psychology: Human Perception and Performance*, *14*(1), 3–11. https://doi.org/10.1037/0096-1523.14.1.3

Titze, C., Heil, M., & Jansen, P. (2008). Gender Differences in the Mental Rotations Test (MRT) Are Not Due to Task Complexity. *Journal of Individual Differences*, *29*(3), 130–133. https://doi.org/10.1027/1614-0001.29.3.130

Voyer, D., & Jansen, P. (2016). Sex differences in chronometric mental rotation with human bodies. *Psychological Research*, *80*(6), 974–984. https://doi.org/10.1007/s00426-015-0701-x

Wiedenbauer, G., Schmid, J., & Jansen-Osmann, P. (2007). Manual training of mental rotation. *European Journal of Cognitive Psychology*, *19*(1), 17–36. https://doi.org/10.1080/09541440600709906
